# Long-term outcome of COVID-19 patients treated with helmet noninvasive ventilation vs. high-flow nasal oxygen: a randomized trial

**DOI:** 10.1186/s40560-023-00669-0

**Published:** 2023-05-19

**Authors:** Teresa Michi, Chiara Mattana, Luca S. Menga, Maria Grazia Bocci, Melania Cesarano, Tommaso Rosà, Maria Rosaria Gualano, Jonathan Montomoli, Savino Spadaro, Matteo Tosato, Elisabetta Rota, Francesco Landi, Salvatore L. Cutuli, Eloisa S. Tanzarella, Gabriele Pintaudi, Edoardo Piervincenzi, Giuseppe Bello, Tommaso Tonetti, Paola Rucci, Gennaro De Pascale, Salvatore M. Maggiore, Domenico Luca Grieco, Giorgio Conti, Massimo Antonelli, for the Gemelli COVID-ICU study group, Riccardo Maviglia, Giovanna Mercurio, Paolo De Santis, Mariano Alberto Pennisi, Gian Marco Anzellotti, Flavia Torrini, Carlotta Rubino, Tony C. Morena, Veronica Gennenzi, Stefania Postorino, Joel Vargas, Nicoletta Filetici, Donatella Settanni, Miriana Durante, Laura Cascarano, Mariangela Di Muro, Roberta Scarascia, Maria Maddalena Bitondo, Martina Murdolo, Alessandro Mele, Serena Silva, Carmelina Zaccone, Francesca Pozzana, Alessio Maccaglia, Martina Savino, Antonella Potalivo, Francesca Ceccaroni, Angela Scavone, Gianmarco Lombardi, Giuseppe Bello, Luca Montini, Antonio Maria Dell’Anna, Carlo Alberto Volta, Vito M. Ranieri, Giulia Falò, Simone Carelli, Daniele Natalini, Cecilia Berardi, Luca Delle Cese, Luigi Vetrugno, Eleonora Balzani

**Affiliations:** 1 Department of Emergency, Intensive Care Medicine and Anesthesia, Fondazione Policlinico Universitario A. Gemelli IRCCS, L.Go F. Vito, 00168 Rome, Italy; 2 Istituto Di Anestesiologia E Rianimazione, Catholic University of The Sacred Heart, Rome, Italy; 3 Department of Hygiene and Public Health, UniCamillus University, Rome, Italy; 4 Department of Anaesthesia and Intensive Care, Infermi Hospital, Rimini, Italy; 5 Department of Morphology, Surgery and Experimental Medicine, Azienda Ospedaliera-Universitaria Arcispedale Sant’Anna, University of Ferrara, Ferrara, Italy; 6 Geriatrics Department, Fondazione Policlinico Universitario A. Gemelli IRCCS, Rome, Italy; 7 Department of Medical and Surgical Sciences, Anesthesia and Intensive Care Medicine, Alma Mater Studiorum, Policlinico Di Sant’Orsola, Università Di Bologna, Bologna, Italy; 8 Department of Biomedical and Neuromotor Science, Alma Mater Studiorum-Università Di Bologna, Bologna, Italy; 9 Department of Anesthesiology, Critical Care Medicine and Emergency, SS. Annunziata Hospital, Chieti, Italy; 10 Leadership in Medicine Research Center, Catholic University of The Sacred Heart, Rome , Italy

**Keywords:** COVID-19, Noninvasive ventilation, Helmet, High-flow nasal oxygen, Acute respiratory failure, Patient self-inflicted lung injury (P-SILI)

## Abstract

**Background:**

Long-term outcomes of patients treated with helmet noninvasive ventilation (NIV) are unknown: safety concerns regarding the risk of patient self-inflicted lung injury and delayed intubation exist when NIV is applied in hypoxemic patients. We assessed the 6-month outcome of patients who received helmet NIV or high-flow nasal oxygen for COVID-19 hypoxemic respiratory failure.

**Methods:**

In this prespecified analysis of a randomized trial of helmet NIV versus high-flow nasal oxygen (HENIVOT), clinical status, physical performance (6-min-walking-test and 30-s chair stand test), respiratory function and quality of life (EuroQoL five dimensions five levels questionnaire, EuroQoL VAS, SF36 and Post-Traumatic Stress Disorder Checklist for the DSM) were evaluated 6 months after the enrollment.

**Results:**

Among 80 patients who were alive, 71 (89%) completed the follow-up: 35 had received helmet NIV, 36 high-flow oxygen. There was no inter-group difference in any item concerning vital signs (*N* = 4), physical performance (*N* = 18), respiratory function (*N* = 27), quality of life (*N* = 21) and laboratory tests (*N* = 15). Arthralgia was significantly lower in the helmet group (16% vs. 55%, *p* = 0.002). Fifty-two percent of patients in helmet group vs. 63% of patients in high-flow group had diffusing capacity of the lungs for carbon monoxide < 80% of predicted (*p* = 0.44); 13% vs. 22% had forced vital capacity < 80% of predicted (*p* = 0.51). Both groups reported similar degree of pain (*p* = 0.81) and anxiety (*p* = 0.81) at the EQ-5D-5L test; the EQ-VAS score was similar in the two groups (*p* = 0.27). Compared to patients who successfully avoided invasive mechanical ventilation (54/71, 76%), intubated patients (17/71, 24%) had significantly worse pulmonary function (median diffusing capacity of the lungs for carbon monoxide 66% [Interquartile range: 47–77] of predicted vs. 80% [71–88], *p* = 0.005) and decreased quality of life (EQ-VAS: 70 [53–70] vs. 80 [70–83], *p* = 0.01).

**Conclusions:**

In patients with COVID-19 hypoxemic respiratory failure, treatment with helmet NIV or high-flow oxygen yielded similar quality of life and functional outcome at 6 months. The need for invasive mechanical ventilation was associated with worse outcomes. These data indicate that helmet NIV, as applied in the HENIVOT trial, can be safely used in hypoxemic patients.

*Trial registration* Registered on clinicaltrials.gov NCT04502576 on August 6, 2020

**Supplementary Information:**

The online version contains supplementary material available at 10.1186/s40560-023-00669-0.

## Background

The need for respiratory support is the most frequent cause of admission to intensive care unit among patients with COVID-19[1]. The optimal first-line approach for respiratory support in acute hypoxemic respiratory failure is debated [2, 3].

In hypoxemic patients, high-flow nasal oxygen is recommended as the first-line intervention due to its effectiveness, accessibility and simplicity of use [4]. Noninvasive ventilation (NIV) with helmet interface and specific settings has been proposed as an alternative tool to manage patients with moderate-to-severe hypoxemia [5–10]. Its putative benefits include the possibility of providing continuous treatments with good tolerability, improved oxygenation, and potential avoidance of lung injury progression [11, 12]. However, its use is limited to highly selected environments with expert personnel, due to the lack of conclusive evidence regarding its efficacy.

Recently, the first head-to-head randomized trial (HENIVOT) compared helmet NIV, eventually followed by high-flow nasal oxygen, versus high-flow oxygen alone as the first-line treatment of patients with COVID-19 and moderate to severe hypoxemic respiratory failure in the intensive care unit [13]. Results showed no significant difference in the number of days free of respiratory support within 28 days from randomization, but a reduced need for endotracheal intubation and invasive mechanical ventilation in the helmet group, suggesting a potential advantage to explore.

Long-term health consequences of COVID-19 have been widely described. These include impaired pulmonary function, physical health and neuropsychological sequelae [14–23]. In patients with acute hypoxemic respiratory failure and acute respiratory distress syndrome, avoidance of endotracheal intubation improves long-term outcome and quality of life [24, 25].

Limited evidence is available regarding the functional outcomes of patients who receive helmet noninvasive ventilation (NIV) in acute hypoxemic respiratory failure [26, 27], and there are concerns about the possible risk of self-inflicted lung injury and pulmonary sequelae due to the effect of spontaneous breathing [28, 29]. To date, no study has ever compared helmet NIV with specific settings vs. high-flow nasal oxygen in terms of long-term outcomes.

In this pre-planned analysis of the HENIVOT trial, we compared the 6-month clinical status, respiratory function, and quality of life of intensive care unit survivors treated with helmet NIV or high-flow nasal oxygen. We also assessed the effects of the need for endotracheal intubation on these outcomes.

## Methods

Patients enrolled in the “HElmet NonInvasive Ventilation versus high-flow Oxygen Therapy in acute hypoxemic respiratory failure (HENIVOT)” were followed up 6 months after randomization. HENIVOT was an investigator-initiated, two-arm, open-label multicentre randomized trial conducted in 4 intensive care units between October and December 2020. The study was supported by the acute respiratory failure study group of the Italian Society of Anesthesia, Analgesia and Intensive Care Medicine (SIAARTI) and was approved by the ethics committee of all participating centres. All patients provided written informed consent for study participation and data analysis.

One hundred-nine patients with moderate-to-severe hypoxemic respiratory failure (ratio of arterial oxygen partial pressure to inhaled oxygen fraction equal to or below 200 mmHg) due to COVID-19 at intensive care unit were randomly assigned in a 1:1 ratio to receive helmet NIV, eventually followed by high-flow nasal oxygen (*N* = 54) or high-flow nasal oxygen alone (*N* = 55). The full study protocol and statistical analysis plan are available elsewhere [13].

### Study design, setting, and participants

All patients discharged alive from hospital were contacted 6 months after enrolment to assess their clinical status, respiratory function, and quality of life. Follow-up visits were performed according to the study protocol from April 2021 to June 2021 in the Italian centers involved in the trial, which are equipped with post-acute outpatient services for individuals discharged from hospital after recovery from COVID-19.

### Variables and data collection

The primary outcome of the study was the percentage of patients with any symptoms at the 6-month follow-up.

Follow-up visits consisted of a global medical assessment including medical history, physical examination, blood tests and pulmonary function test.

Patients were interviewed face-to-face with a set of questionnaires that measure physiological and psychological status, including: the modified British Medical Research Council (mMRC, a clinical tool to stratify dyspnea) [30], the EuroQoL five dimensions five levels (EQ-5D-5L) questionnaire (an instrument to measure health-related quality of life) [31], the EuroQol Visual Analogue Scale (EQ-VAS, a self-rated health record) [31], the Medical Outcomes Study 36 Items Short form (SF-36, a patient-report questionnaire on quality of life) [32, 33], the Post Traumatic Stress Disorder Checklist for the Diagnostic and Statistical Manual of mental disorder (DMS-5) (PCL-5, that assesses the symptoms of post-traumatic stress disorder) [34] and a self-reported symptoms questionnaire (Additional file 4: Material, E-Appendix).

The physical examination included the six minutes walking test [35] and 30-s chair stand test [36].

Venous blood samples were collected and analyzed to determine complete blood cell count (hemoglobin, platelet count, white blood cell count), hepatic function (total bilirubin and transaminases) and renal function (creatinine, blood urea nitrogen and estimated glomerular filtration rate).

Pulmonary function test consisted of global spirometry with diffusion capacity for carbon monoxide (DLCO) assessment and arterial blood gas analysis.

All data were recorded in an electronic database.

### Statistical analysis

Categorical variables were summarized as absolute and percentage frequencies and continuous variables as median and interquartile range. Between-group comparisons were performed using Fisher’s or Chi-square test for categorical variables, Mann–Whitney test for ordinal variables or non-normal quantitative variables and *t* test for normally distributed quantitative variables.

To estimate the effect of endotracheal intubation on physical and psychological performance status at 6 months, we compared the study variables between patients who required endotracheal intubation and who did not require endotracheal intubation, independently from the assigned treatment.

Some patients had missing data for some outcomes: the exact number of patients with complete data for a determined group of variables is specified in the tables. Given that the data were missing at random, no imputation was performed. The significance level was set at *p* < 0.05. All statistical tests were two-tailed.

We performed a Random Forest analysis and the respective MDS plot with as independent variables the allocation treatment and the endotracheal intubation, to explore their effects on a number of outcomes (Additional file 1–2). The model was trained to optimize the number of trees.

Statistical analysis was performed with SPSS (IBM Corp. Released 2019. IBM SPSS Statistics for Windows, Version 26.0. Armonk, NY), GraphPad Prism (GraphPad Software, San Diego, California USA, version 7.0.0 for Windows) and R software (Rstudio Team (2020). RStudio: integrated Development for R. RStudio, PBC, Boston, Version 2023.03.0 + 386).

## Results

**Fig. 1:**
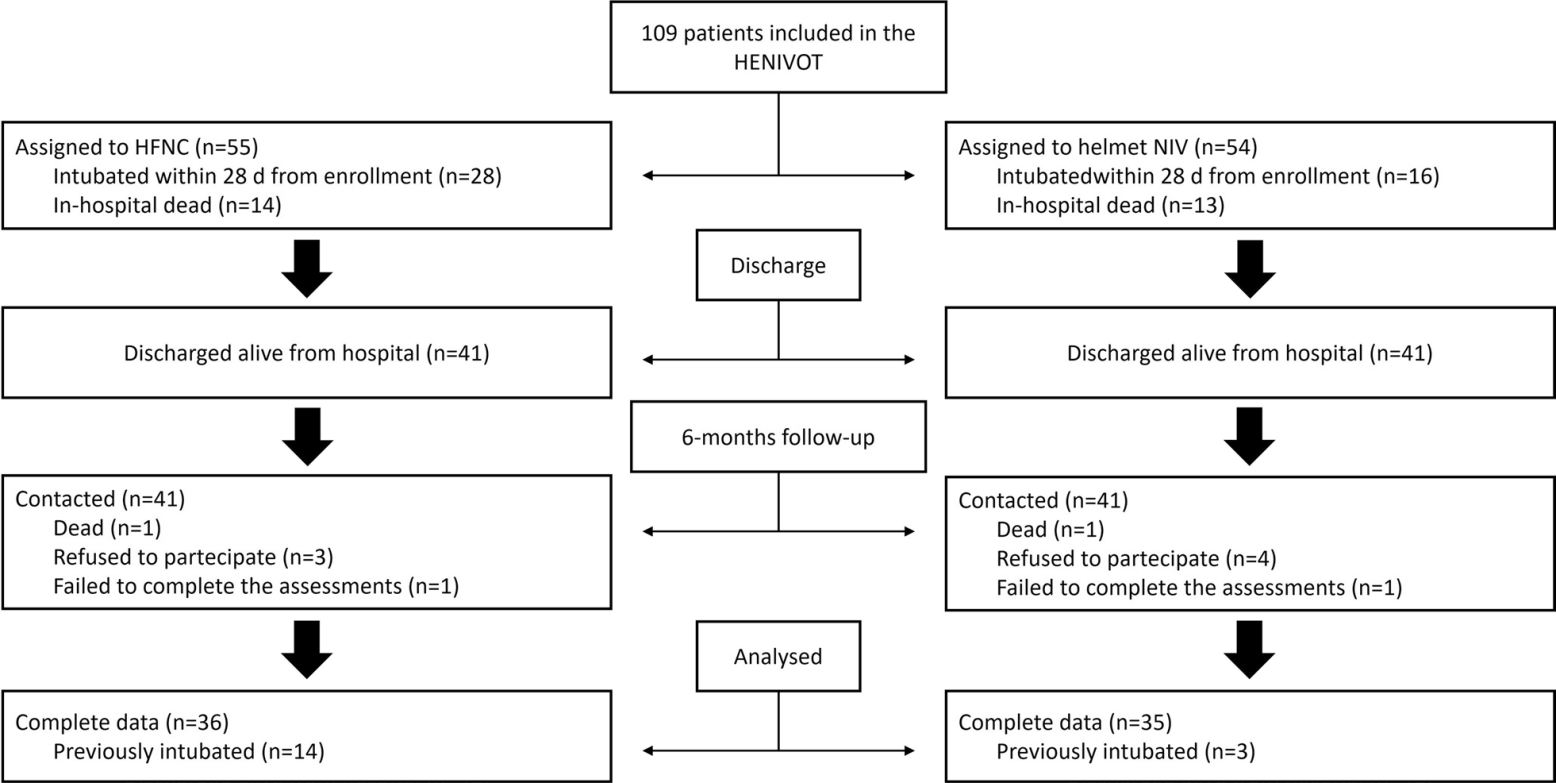
Study flow-chart

Eighty-two of the 109 patients enrolled in the HENIVOT trial were discharged alive from the hospital and were contacted at 6 months; among these patients, 2 were deceased, 7 refused to participate and 2 were lost at follow-up. Of remaining 71, who successfully completed the follow-up, 35 received helmet NIV and 36 high-flow oxygen (Fig. 1). Table 1 shows the patient’s demographics.

**Table 1:** Characteristics of patients, according to the study group.*

Characteristic	Helmet noninvasive ventilation (*N* = 35)	High-flow nasal oxygen (*N* = 36)	*P* value
Age (years)	67 [53–73]	59 [53–69]	0.10
Female sex—*N* (%)	9 (26)	4 (11)	0.14
Body Mass index^a^	27 [26–30]	28 [25–32]	0.94
Most relevant comorbidities^b^			
Hypertension—*N* (%)	16 (46)	18 (50)	0.81
Type 2 diabetes mellitus—*N* (%)	9 (26)	5 (14)	0.25
Smoking—*N* (%)	11 (31)	17 (47)	0.23
Immunocompromised state—*N* (%)	3 (9)	3 (8)	> 0.99
Recent chemotherapy—*N* (%)	1 (3)	0 (0)	–
Human Immunodeficiency Virus—*N* (%)	1 (3)	0 (0)	–
Immunosuppressive therapy–renal transplant—*N* (%)	0 (0)	1 (3)	–
Acute myeloid leukemia—*N* (%)	0 (0)	1 (3)	–
Ulcerative colitis-immunosuppressive therapy—*N* (%)	0 (0)	1 (3)	–
History of cancer—*N* (%)	4 (11)	0 (0)	0.05
Neurological conditions—*N* (%)	0 (0)	0 (0)	–
SAPS II^c^	32 [24–35]	27 [24–32]	0.04
SOFA^d^	2 [2–3]	2 [2–3]	0.46
Bilateral lung injury^e^—*N* (%)	35 (100)	36 (100)	
Intubation during COVID hospitalization—*N* (%)	3 (9)	14 (39)	0.005
Respiratory support free days	22 [15–25]	21 [11–23]	0.24
Hours to endotracheal intubation	52 [5–70]	11 [3–23]	0.16
Invasive ventilation-free days			
28 days	28 [28–28]	28 [17–28]	0.003
60 days	60 [60–60]	60 [49–60]	0.004
Duration of stay, days			
Intensive care unit	7 [3–10]	8 [4–16]	0.26
Hospital	18 [14–27]	19 [13–38]	0.75
Tracheostomy—*N* (%)	1 (3)	7 (19)	0.05
Rescue therapies			
Paralysis—*N* (%)	3 (9)	13 (36)	0.13
Prone positioning—*N* (%)	3 (9)	10 (28)	0.42
Extracorporeal membrane oxygenation—*N* (%)	0 (0)	3 (8)	0.24

*Values are displayed as medians [interquartile range], if not otherwise specified

^a^The body-mass index is the weight in kilograms divided by the square of the height in meters

^b^Medical history was obtained from the patient and the medical record

^c^SAPS II was calculated from 17 variables at enrollment, information about previous health status, and information obtained at admission. Scores range from 0 to 163, with higher scores indicating more severe disease

^d^SOFA score was calculated from 6 variables at enrollment, information about previous health status, and information obtained at admission. Scores range from 0 to 24, with higher scores indicating more severe disease

^e^All patients received chest X-ray the day of enrollment

### Symptoms and physical performance test

There was no difference in the primary outcome: the majority of patients reported at least one symptom at the follow-up: 28 (90%) in the helmet group and 28 (85%) in the high-flow group (*p* = 0.71). Overall, the most common symptoms were dyspnea and fatigue, with a median mMRC, respectively, of 2 [IQR: 1–2] in the helmet group vs. 2 [IQR: 2–3] in the high-flow group (*p* = 0.25). Patients in the helmet group reported a significantly lower frequency of arthralgia, meant as joint pain or stiffness (16% vs. 55%, OR = 0.16 [95% CI 0.05–0.52], (*p* = 0.002). No significant difference between groups was found in the incidence of dyspnea, fatigue, dry cough, sore throat, rhinitis, smell and taste disorders and decreased visual acuity (Table 2).

**Table 2:** Outcomes at 6 months, according to study group*

	Helmet noninvasive ventilation (*N* = 31)	High-flow nasal oxygen (*N* = 33)	Absolute or mean difference (95% CI)	Odds ratio (95% CI)	*P* value
Symptoms					
Anyone of the following symptoms—*N* (%)	28 (90)	28 (85)	5 (− 11 to 22)	1.67 (0.36 to 7.65)	0.71
Fatigue—*N* (%)	17 (55)	18 (55)	0 (− 0.23 to 0.23)	1.01 (0.38 to 2.71)	> 0.99
Dyspnea—*N* (%)	22 (71)	21 (64)	7 (− 15 to 29)	1.4 (0.49 to 4.00)	0.60
mMRC scale (range 0–4)	2 [1–2]	2 [2–3]	0.17 (− 0.16 to 0.51)		0.25
Dry cough—*N* (%)	2 (6)	3 (9)	− 03 (− 18 to 13)	0.69 (0.11 to 4.43)	> 0.99
Sore throat—*N* (%)	0 (0)	2 (6)	− 6 (− 20 to 6)	-	0.49
Productive cough—*N* (%)	0 (0)	0 (0)	0 (− 0 to 11)	-	-
Rhinitis—*N* (%)	2 (6)	1 (3)	3 (− 10 to 18)	2.21 (0.19 to 25.64)	0.61
Smell disorder—*N* (%)	3 (10)	1 (3)	7 (− 7 to 22)	3.43 (0.34 to 34.86)	0.35
Decreased visual acuity– *N* (%)	5 (16)	2 (6)	10 (− 6 to 27)	2.98 (0.53 to 16.66)	0.25
Conjunctival hyperaemia—*N* (%)	0 (0)	0 (0)	0 (− 0 to 11)	-	-
Taste disorder—*N* (%)	2 (6)	2 (6)	0 (14 to 15)	1.07 (0.14 to 8.09)	> 0.99
Inappetence—*N* (%)	1 (3)	2 (6)	− 3 (− 17 to 11)	0.52 (0.04 to 6.00)	> 0.99
Diarrhoea—*N* (%)	4 (13)	0 (0)	13 (0 to 29)	-	0.05
Myalgia—*N* (%)	9 (29)	12 (36)	− 7 (− 29 to 15)	0.72 (0.25 to 2.05)	0.60
Arthralgia—*N* (%)	5 (16)	18 (55)	− 38 (− 56 to − 15)	0.16 (0.05 to 0.52)	0.002
Chest pain—*N* (%)	3 (10)	2 (6)	4 (− 11 to 9)	1.66 (0.26 to 10.68)	0.67
Sicca syndrome—*N* (%)	1 (3)	1 (3)	0 (− 12 to 13)	1.07 (0.06 to 17.83)	> 0.99
Raynaud syndrome—*N* (%)	0 (0)	0 (0)	0 (− 10 to 11)	-	-
Skin lesion—*N* (%)	0 (0)	0 (0)	0 (− 10 to 11)	-	-
Syncope—*N* (%)	0 (0)	0 (0)	0 (− 10 to 11)	-	-
Dizziness—*N* (%)	1 (3)	0 (0)	3 (− 8 to 16)	-	0.48
Headache—*N* (%)	0 (0)	0 (0)	0 (− 10 to 11)	-	-

Physical performance test					
Pulmonary performance test	Helmet noninvasive ventilation (*N* = 30)	High-flow nasal oxygen (*N* = 30)	Absolute or mean difference (95% CI)	Odds ratio (95% CI)	*P* value
Forced vital capacity % of predicted	92 [84–104]	88 [80–98]	− 6.03 (− 13.96 to 1.88)		0.22
Forced vital capacity < 80% of predicted—*N* (%)	4 (13)	7 (22)	− 9 (− 27 to 11)	0.55 (0.14 to 2.11)	0.51
Forced expiratory volume in one second % of predicted	94 [87–108]	93 [80–102]	− 4.95 (− 12.8 to 2.90)		0.31
Forced expiratory volume in one second < 80% of predicted—*N* (%)	3 (10)	7 (23)	− 13 (− 31 to 7)	0.38 (0.09 to 1.64)	0.30
Forced expiratory volume in one second/Forced vital capacity ratio	0.81 [0.77–0.85]	0.82 [0.79–0.85]	0.02 (− 0.01 to 0.05)		0.47
Forced expiratory volume in one second/Forced vital capacity ratio < 80% of predicted—*N* (%)	7 (23)	11 (37)	− 14 (− 35 to 9)	1.99 (0.65 to 6.10)	0.27
Maximal (mid-) expiratory flow 25–75—litres per second	1.13 [0.97–1.33]	1.16 [0.95–1.37]	0.02 (− 0.15 to 0.19)		0.95
Total lung capacity % of predicted	90 [85–98]	88 [77–92]	− 6.22 (− 13.22 to 0.88)		0.12
Total lung capacity < 80% of predicted—*N* (%)	4 (13)	10 (33)	− 20 (− 40 to 2)	0.31 (0.08 to 1.13)	0.13
Diffusing capacity of the lung for carbon monoxide % of predicted	78 [70–85]	76 [67–88]	− 1.41 (− 10.43 to 7.60)		0.67
Diffusing capacity of the lung for carbon monoxide < 80% of predicted—*N* (%)	15 (52)	19 (63)	− 0.12 (− 0.34 to 0.13)	0.62 (0.22 to 1.76)	0.44
Alveolar ventilation—litres	5.41 [4.32–5.99]	5.45 [4.29–5.86]	0.11 (− 0.50 to 0.72)		0.95
Diffusing capacity of the lung for carbon monoxide/alveolar ventilation ratio	0.94 [0.84–1.02]	0.96 [0.86–1.04]	0.02 (− 0.08 to 0.11)		0.64
Residual volume % of predicted	84 [75–97]	84 [69–91]	− 5.33 (− 15.05 to 4.39)		0.41
Residual volume < 80% of predicted—*N* (%)	11 (37)	11 (35)	2 (− 22 to 24)	1.05 (0.37 to 2.99)	> 0.99
Residual volume/total lung capacity ratio % of predicted	90 [83–101]	90 [83–99]	− 0.1 (− 6.42 to 6.22)		0.96

Six minutes walking test	Helmet noninvasive ventilation (*N* = 30)	High-flow nasal oxygen (*N* = 30)	Absolute or mean difference (95% CI)	Odds ratio (95% CI)	*P* value
Distance walked—metres	490 [420–540]	510 [438–540]	− 5.23 (− 56.63 to 46.17)		0.82
Percentage of predicted value—%	127 [115–150]	125 [105–144]	− 8.61 (− 24.23 to 7.01)		0.36
Less than lower limit of the predicted value—*N* (%)	1 (4)	4 (15)	− 10 (− 28 to 7)	0.20 (0.02 to 1.96)	0.18
SpO2 nadir during test—%	94 [91–95]	94 [91–95]	− 0.006 (− 0.02 to 0.01)		0.50
Recovery time to return SpO2 to basal value—minutes	1 [1–2]	2 [1–2]	0.36 (− 0.16 to 0.88)		0.43
Maximum heart rate—beats per minutes	107 [99–116]	103 [99–112]	− 2.17 (− 9.01 to 4.68)		0.46
Recovery time to return heart rate to basal value—minutes	2 [2–3]	2 [2–3]	0.23 (− 0.27 to 0.72)		0.36
BORG scale for dyspnea during test (range 0–10)	4 [2–6]	3 [3–5]	− 0.24 (− 1.43 to 0.95)		0.91
Interruption of test—*N* (%)	0 (0)	1 (3)	− 3 (− 17 to 8)	-	0.49

Quality-of-life assessment	Helmet noninvasive ventilation (*N* = 35)	High-flow nasal oxygen (*N* = 36)	Absolute or mean difference (95% CI)	Odds ratio (95% CI)	*P* value
EQ-VAS (range 0–100)	78 [69–80]	70 [60–80]	− 4.67 (− 11.39 to 2.05)		0.27
EQ-5D-5L					
Impairment in mobility (mobility > 1)—*N* (%)	10 (29)	14 (39)	− 10 (− 31 to 11)	0.63 (0.23 to 1.70)	0.45
Impairment in personal care (personal care > 1)—*N* (%)	7 (20)	8 (22)	− 2 (− 21 to 17)	0.88 (0.28 to 2.74)	> 0.99
Impairment in usual activities (usual activities > 1)—*N* (%)	11 (31)	17 (47)	− 16 (− 36 to 7)	0.51 (0.20 to 1.35)	0.23
Reported pain or discomfort (pain or discomfort > 1)—*N* (%)	19 (54)	21 (58)	− 4 (− 26 to 18)	0.85 (0.33 to 2.17)	0.81
Reported anxiety or depression (anxiety or depression > 1)—*N* (%)	12 (34)	14 (39)	− 5 (− 26 to 17)	0.82 (0.31 to 2.16)	0.81
PCL-5 (range 0–100)	11 [3–17]	8 [2–17]	0.07 (− 7.37 to 7.51)		0.71
SF-36					
Physical functioning (range 0–100)	85 [39–95]	78 [34–95]	− 4.11 (− 20.00 to 11.78)		0.53
Limitations due to physical health (range 0–100)	50 [0–100]	50 [0–100]	− 3.53 (− 24.61 to 17.54)		0.68
Limitations due to emotional problems (range 0–100)	67 [25–100]	100 [33–100]	7.65 (− 12.79 to 28.08)		0.41
Energy/fatigue (range 0–100)	60 [40–75]	60 [36–74]	− 2.75 (− 13.25 to 7.76)		0.56
Emotional well-being (range 0–100)	76 [60–88]	78 [61–88]	1.09 (− 8.50 to 10.68)		0.90
Social functioning (range 0–100)	88 [63–100]	75 [41–88]	− 7.80 (− 20.56 to 4.97)		0.16
Pain (range 0–100)	85 [55–100]	78 [55–90]	− 4.35 (− 16.50 to 7.79)		0.33
General health (range 0–100)	60 [45–75]	55 [35–74]	− 3.12 (− 14.12 to 7.88)		0.52
Health change (range 0–100)	25 [25–50]	38 [25–50]	2.44 (− 10.89 to 15.77)		0.72

*Values are displayed as medians [interquartile range], if not otherwise specified

Vital signs, laboratory tests and arterial blood gas were similar between the two groups and did not present significant abnormalities (Additional file 3: Table S1).

**Fig. 2:**
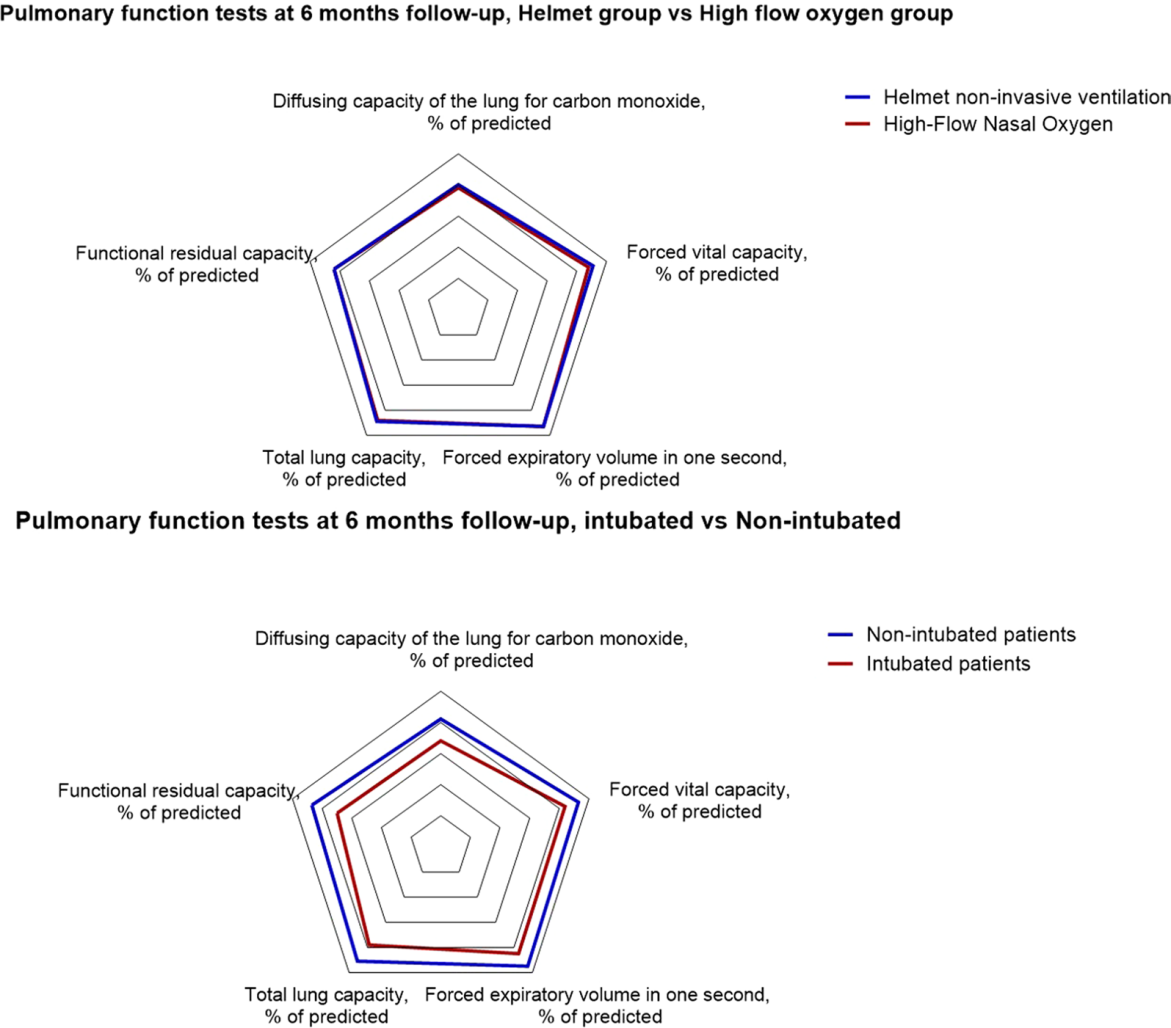
Radar chart showing pulmonary function tests at 6 months in our cohort. Both patients treated with Helmet NIV and high-flow had reduced performance at 6 months, but there was no difference between the two groups (upper panel). Patients that did not require endotracheal intubation had pulmonary function tests at 6 months more similar to physiological values, compared to patients that required endotracheal intubation (lower panel)

Pulmonary function parameters were impaired in the overall cohort, with no significant differences between groups (Fig. 2, Table 2, Additional file 3: Table S2). DLCO impairment was the most frequent abnormality: 78% [70–85] of the predicted value among patients treated with helmet and 76% [67–88] among those treated with high-flow nasal oxygen (*p* = 0.67).

Median distance at six minutes walking test was 490 m [IQR: 420–540] in the helmet group and 510 m [IQR: 438–540] in the high-flow group (*p* = 0.82), without significant differences in terms of dyspnea assessed by the BORG scale (4 [IQR: 2–6] vs. 3 [IQR: 3–5], respectively, *p* = 0.91). One (4%) patient in the helmet group vs. 4 (15%) in the high flow group were unable to walk the predicted distance (*p* = 0.18).

The 30-s chair stand test was successfully completed by all participants, with 23 cycles in 30 s [20–29] in the helmet group and 23 [19–25] in the high flow group (*p* = 0.65). (Additional file 3: Table S2).

### Health-related quality-of-life assessment

Full details of the EQ-VAS, EQ-5D-5L, PCL5, SF-36 questionnaires are presented in Table 2 and in Additional file 3: Table S2.

**Fig. 3:**
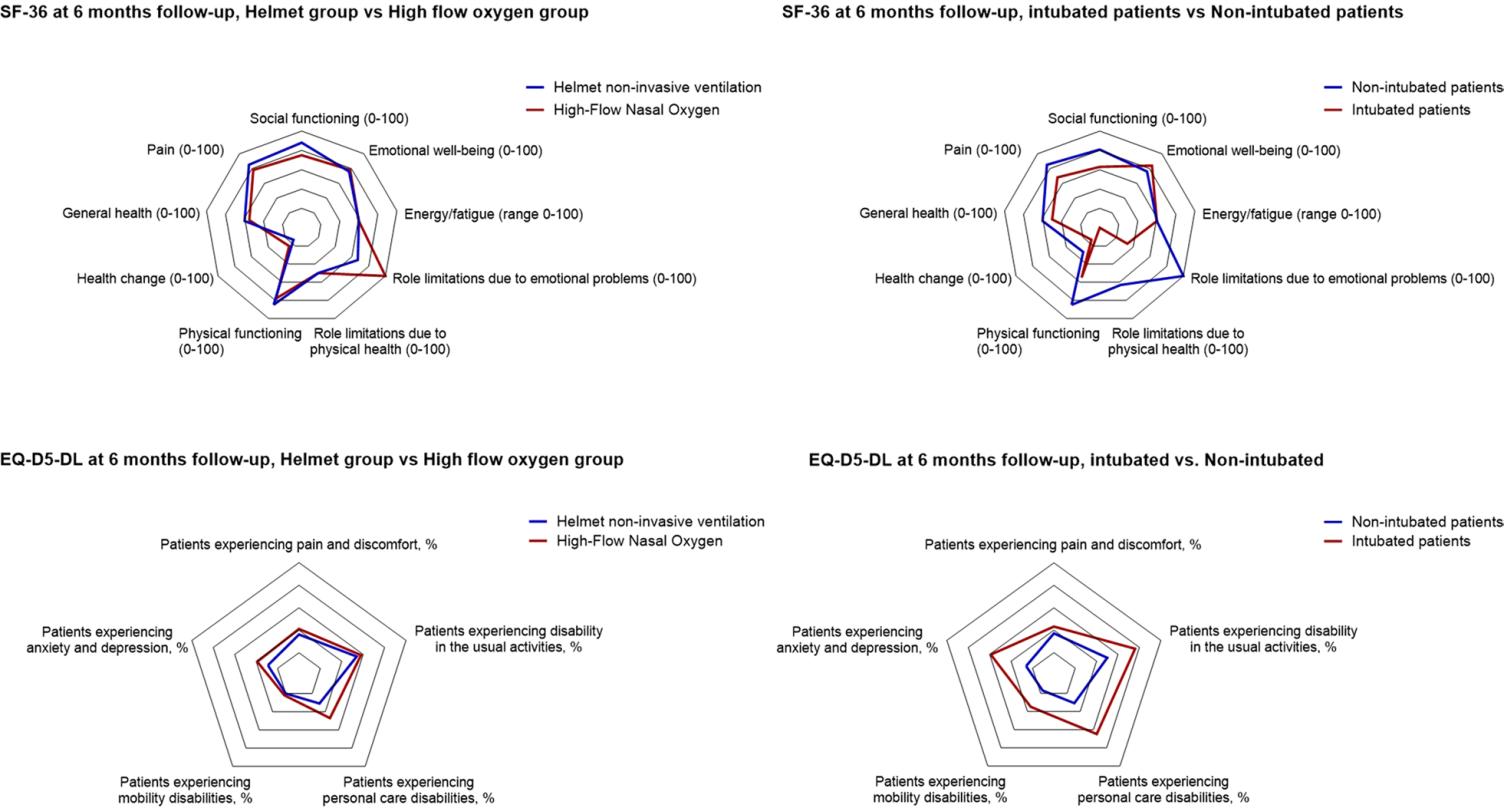
Radar chart showing health-related quality-of-life assessment in our cohort. For the SF-36 test the higher score represents a better subjective health experience. The top left panel shows no difference in subjective health experience at 6 months between the high-flow group and the helmet group; however, patients that required endotracheal intubation reported worse subjective health experience compared to those who avoided endotracheal intubation (top right panel). The lower panel shows the EuroQoL five dimension five levels (EQ-5D-5L). The radar chart shows the percentage of patients reporting problems in each of the five dimensions. There was no difference between the high-flow and the helmet group (bottom left panel); however, a higher percentage of patients that needed endotracheal intubation reported impairment in each of the five dimensions, compared to the patients that avoided endotracheal intubation (bottom right panel)

The two groups had similar SF-36 scores in all domains; the most impaired domains were “role limitation due to physical health”, “general health” and “health change”, without differences between groups (all *p* > 0.05) (Fig. 3, Table 2).

Many patients reported decreased quality of life in the EQ-5D-5L, including pain (21 patients (58%) in the high-flow oxygen group vs. 19 (54%) vs. the helmet group, *p* = 0.81) and anxiety or depression (14 patients (39%) in the high-flow oxygen group vs. 12 (34%) in the helmet group, *p* = 0.81) (Fig. 3).

The EQ-VAS score was 70 [60–80] in the high-flow group vs. 78 [69–80] in the helmet group (*p* = 0.27). The median PCL-5, assessing post-traumatic stress disorder, did not differ significantly between the two groups (8 [2–17] vs. 11 [3–17], *p* = 0.71), and in the majority of the cohort it was far below the cutoff score of 33 that defines post-traumatic stress disorder.

### Effect of endotracheal intubation on outcomes

We compared 17 patients who required endotracheal intubation (3 on Helmet NIV, 14 on HFNC) and 54 patients who did not (32 on Helmet NIV, 22 on HFNC). Besides the type of respiratory support, no other significant differences were found between patients who required endotracheal intubation vs. those who did not (Additional file 3: Table S3).

Patients who required endotracheal intubation, compared to those who did not, showed a higher incidence of fatigue (13 (76%) vs. 22 (47%), OR = 3.69 [95%CI 1.05–13.00], *p* = 0.05), arthralgia (11 (65%) vs. 12 (26%), OR = 5.35 [95%CI 1.63–17.60], *p* = 0.007) (Table 3) and decreased estimated glomerular filtration rate (80 [58–91] vs. 92 [IQR 78–101] ml/min/1.73m^2^, mean difference 14.44 [95%CI 0.80–28.09], *p* = 0.04) (Additional file 3: Table S4).

**Table 3:** Outcomes at 6 months according to intubation*

Symptoms	Non-intubated patients (*N* = 47)	Intubated patients (*N* = 17)	Absolute or mean difference (95% CI)	Odds ratio (95% CI)	*P* value
Any symptom—*N* (%)	39 (83)	17 (100)	− 17 (− 29 to 10)	–	0.10
Most frequent symptoms§					
Fatigue—*N* (%)	22 (47)	13 (76)	− 30 (− 49 to − 2)	3.69 [1.05 to 13.00]	0.05
Arthralgia—*N* (%)	12 (26)	11 (65)	− 39 (− 60 to − 12)	5.35 [1.63 to 17.60]	0.007

Physical performance test					
Pulmonary function test	Non-intubated patients (*N* = 45)	Intubated patients (*N* = 15)	Absolute or mean difference (95% CI)	Odds ratio (95% CI)	*P* value
Forced vital capacity % of predicted	93 [84–104]	84 [69–92]	14.53 (5.88 to 23.17)		0.009
Forced vital capacity < 80% of predicted—*N* (%)	5 (11)	6 (40)	− 29 (− 54 to − 6)	5.60 [1.40 to 22.44]	0.02
Forced expiratory volume in one second /Forced vital capacity ratio	0.81 [0.77–0.84]	0.85 [0.81–0.88]	− 4 (− 8 to 0)		0.03
Total lung capacity % of predicted	91 [85–98]	78 [62–90]	14.89 (7.54 to 22.23)		0.001
Total lung capacity < 80% of predicted—*N* (%)	6 (13)	8 (53)	− 40 (− 63 to − 13)	7.43 [1.97 to 28.08]	0.003
Diffusing capacity of the lung for carbon monoxide % of predicted	80 [71–88]	66 [47–77]	16.40 (6.51 to 26.29)		0.005
Diffusing capacity of the lung for carbon monoxide < 80% of predicted—*N* (%)	22 (49)	12 (86)	− 37 (− 54 to − 8)	6.27 [1.26 to 31.29]	0.03
Residual volume % of predicted	87 [78–96]	70 [58–85]	15.95 (5.42 to 26.49)		0.006
Residual volume < 80% of predicted—*N* (%)	14 (30)	8 (53)	− 23 (− 48 to 4)	2.61 [0.79 to 8.61]	0.13
Residual volume/total lung capacity ratio % of predicted	90 [83–101]	90 [80–96]	2.78 (− 4.48 to 10.03)		0.68

Six minutes walking test	Non-intubated patients (*N* = 46)	Intubated patients (*N* = 14)	Absolute or mean difference (95% CI)	Odds Ratio (95% CI)	*P* value
Distance walked—metres	510 [450–540]	465 [388–528]	51.84 (− 7.40 to 111.07)		0.15
Percentage of predicted value—%	128 [112–149]	122 [99–130]	20.21 (1.43 to 38.99)		0.13
Less than lower limit of the predicted value—*N* (%)	2 (5)	3 (27)	− 23 (− 52 to − 2)	7.69 [1.10 to 53.65]	0.05

Quality-of-life assessment	Non-intubated patients (*N* = 54)	Intubated patients (*N* = 17)	Absolute or mean difference (95% CI)	Odds Ratio (95% CI)	*P* value
EQ-VAS (range 0–100)	80 [70–83]	70 [53–70]	9.26 (1.89 to 16.64)		0.01
EQ-5D-5L					
Impairment in mobility (mobility > 1)—*N* (%)	14 (26)	10 (59)	− 33 (− 55 to − 7)	4.08 (1.30 to 12.78)	0.02
Impairment in personal care (personal care > 1)—*N* (%)	9 (17)	6 (35)	− 19 (− 43 to 3)	2.73 (0.80 to 9.29)	0.17
Impairment in usual activities (usual activities > 1)—*N* (%)	17 (31)	11 (65)	− 33 (− 54 to − 6)	3.99 (1.27 to 12.58)	0.02
Reported pain or discomfort (pain or discomfort > 1)—*N* (%)	27 (50)	13 (76)	− 26 (− 45 to 1)	3.25 (0.94 to 11.24)	0.09
Reported anxiety or depression (anxiety or depression > 1)—*N* (%)	19 (35)	7 (41)	− 6 (− 31 to 18)	1.29 (0.42 to 3.94)	0.77
PCL-5 (range 0–100)	7 [2–16]	14 [4–27]	− 5.32 (− 14.03 to 3.40)		0.10
SF-36					
Physical functioning (range 0–100)	85 [44–95]	55 [20–78]	19.38 (1.32 to 37.44)		0.01
Limitations due to physical health (range 0–100)	63 [0–100]	0 [0–63]	23.67 (− 0.38 to 47.71)		0.05
Limitations due to emotional problems (range 0–100)	100 [33–100]	33 [0–100]	14.46 (− 9.33 to 38.25)		0.26
Energy/fatigue (range 0–100)	60 [40–75]	60 [28–70]	8.27 (− 3.90 to 20.44)		0.23
Emotional well-being (range 0–100)	76 [63–88]	84 [54–90]	1.16 (− 10.08 to 12.40)		0.88
Social functioning (range 0–100)	81 [50–100]	63 [38–94]	10.24 (− 4.67 to 25.16)		0.21
Pain (range 0–100)	85 [55–100]	68 [39–89]	13.76 (− 0.13 to 27.65)		0.03
General health (range 0–100)	60 [45–75]	50 [33–75]	2.86 (− 10.04 to 15.76)		0.57
Health Change (range 0–100)	50 [25–50]	25 [25–38]	15.66 (0.48 to 30.83)		0.05

*Values displayed are medians and interquartile range if not otherwise specified

^§^All symptoms are reported in Additional file 3: Table S4

Compared to patients who successfully avoided intubation with noninvasive support, a higher proportion of intubated patients showed DLCO < 80% (OR = 6.27 [95%CI 1.26–31.29], *p* = 0.03), forced vital capacity < 80% (OR = 5.6 [95%CI 1.40–22.44], *p* = 0.02) and total lung capacity (OR = 7.43 [95%CI 1.97–28.08], *p* = 0.003) (Table 3, Additional file 3: Table S4, Fig. 3).

Median EQ-VAS was significantly lower in intubated patients, compared to those who had not (70 [53–70] vs. 80 [70–83], with a mean difference of 9.26 [95%CI 1.89–16.64], *p* = 0.01) (Fig. 3, Table 3, Additional file 3: Table S4).

Compared to non-intubated patients, a higher proportion of intubated patients reported mild problems (EQ-D5-L5 > 1) doing usual activities of the daily living (65% vs. 31%, OR = 3.99 [95%CI 1.27–12.58], *p* = 0.02) and in the mobility (59% vs. 26%, OR = 4.08 [95%CI 1.30–12.78], *p* = 0.02).

Compared to non-intubated patients, patients who had received invasive mechanical ventilation reported lower SF-36 scores, showing limitations in physical functioning (*p* = 0.01), limitations due to decreased physical health (*p* = 0.05), chronic pain (*p* = 0.03), and worsen overall health (*p* = 0.05) (Fig. 3, Table 3).

## Discussion

The results of our study on the functional outcomes of patients affected by COVID-19 moderate-to-severe respiratory failure treated with helmet noninvasive ventilation vs. high-flow nasal oxygen can be summarized as follows:

- The incidence of symptoms was high in both groups, with no significant difference between them, except for a lower incidence of arthralgia in the helmet group.
- Both groups demonstrated decreased physical status, functionality for pulmonary tests, and quality of life.
- Patients who required endotracheal intubation had worse physical and psychological outcome at 6 months compared to those successfully treated with non-invasive respiratory support.

Exploring long-term consequences of these treatments is crucial to inform the ongoing debate regarding the optimal non-invasive management of acute respiratory failure.

Preserving spontaneous breathing in moderate-to-severe hypoxemic patients has benefits and risks.

It can help avoid complications related to invasive mechanical ventilation and prevent diaphragm dysfunction [28, 37], but may also exacerbate lung injury due to the combination of dysregulated respiratory drive and lung inhomogeneities causing patient self-inflicted lung injury (P-SILI) [29, 38–40].

P-SILI can occur during any noninvasive support strategy, but most concerns exist when mechanical increases in airway pressure are applied during NIV [41, 42]. In this context, high PEEP (of at least 10 cmH_2_O) can reduce lung inhomogeneity, improve lung mechanics, and decrease inspiratory effort, potentially mitigating the detrimental effects related to P-SILI [43–46]. However, if the inspiratory effort is low, helmet NIV leads to increased transpulmonary pressure and tidal volumes, which possibly worsen outcomes [41, 44, 47]. These risks underscore the importance of examining long-term outcomes, to rule out possible adverse effects on lung function caused by noninvasive support.

In our study, a considerable proportion of severe COVID-19 survivors experienced persistent symptoms, decreased respiratory performance and reduced quality of life 6 months after the infection, with no significant difference between helmet NIV and high-flow group. This overall rate of long-term sequalae reported in our study is consistent with current evidence [17, 20–23, 48, 49]. The only statistically significant difference between groups was an increased incidence of arthralgia in the high-flow group (55% vs. 16%, *p* = 0.002). Arthralgia is one of the most common long-term symptoms of COVID-19 [50]. The underlying pathophysiological mechanism is not fully understood, but likely involves biochemical and inflammatory response pathways, such as cytokine increase and dysregulation [51]. The higher incidence of arthralgia in the high-flow group may reflect the more frequent use of invasive mechanical ventilation, prone position sessions and muscle paralysis in these patients. Notably, all intubated patients received at least one session of prone positioning and muscle paralysis for a minimum of 24–48 h.

A significant proportion of patients had respiratory impairment 6 months after COVID-19, which is consistent with previous findings [19, 52–58]. Decreased diffusing capacity and gas exchange impairment, reported in over half of the patients, are related to pulmonary interstitial damage [59]. This may be linked to the reduction of pulmonary volumes, which was detected in up to one-third of our patients without difference between study groups: this may suggest a fibrotic evolution of the disease [60, 61].

Regarding health-related quality-of-life impairment, our findings align with other investigations and are linked to the psychological stress caused by intensive care unit stay, respiratory support and extended hospitalization and isolation periods [17, 48, 49, 56, 62]. Despite the general psychological impairment, our cohort did not present post-traumatic stress disorder symptoms, such as sleeping and behavioral issues, according to the PCL-5 test.

Health-related quality of life of patients treated with helmet NIV was also explored by the recent follow-up study of the Helmet-COVID randomized trial [27]. Consistently with our results, they found no difference in the EQ-VAS and the EQ-5D-5L questionnaire between helmet NIV and usual respiratory support: however, in the Helmet-COVID study, the control group included patients treated with high-flow oxygen, facemask NIV and conventional oxygen according to the clinical practice of participating centers. Differently, our study compared helmet NIV vs. high-flow nasal oxygen, which is the suggested first-line treatment for hypoxemic respiratory failure: this enhances the external validity and reproducibility of our findings.

Despite the difference in endotracheal intubation rate (9% in the helmet group vs. 28% in the high-flow group, *p* = 0.005), we did not find significant effects on long-term outcomes. This finding may be considered as surprising, since previous reports showed that avoiding intubation yields improved long-term outcomes, while endotracheal intubation, sedation, paralysis and invasive mechanical ventilation are associated with higher mortality and morbidity [24, 25, 63]. Lung protective ventilation bundles (including delivery of low tidal volumes and extensive use of prone position) and prevention measures for intensive care unit-acquired infections, which were rigorously applied in all patients. These may have contributed to limit the detrimental effects of invasive mechanical ventilation among patients who survived [37], mitigating any inter-group difference between patients treated with helmet NIV or high-flow oxygen.

To address this aspect, we conducted a secondary analysis, comparing study outcomes for patients who received endotracheal intubation and invasive ventilation vs. those who did not. We found significant impairment in most items concerning pulmonary function, physical performance, and quality of life in patients who had been intubated vs. those who had not (Table 3, Figs. 2 and 3).

Furthermore, we performed two Random Forest analyses and display the respective MDS plot: one with treatment using high-flow or helmet NIV as independent variables and the other with endotracheal intubation as the independent variable. While the treatment allocation had a high out-of-bag estimation rate (39%), yielding higher uncertainties in its predictive power, a cluster of a group of patients treated with high-flow is identifiable in the confusion matrix (Additional file 1: Figure S1a). Despite exploratory in nature, this finding suggests a possible protective effect of helmet NIV on analyzed outcomes. Conversely, endotracheal intubation had a lower out-of-bag estimation rate (19.72%), suggesting lower uncertainties in its predictive power. In this case, although clustering could not be observed (Additional file 1 and 2: Figure S1a–b), a clear asymmetry in the model is presented, suggesting a strong relationship between endotracheal intubation and our outcomes.

Our study has relevant clinical implications: while avoiding endotracheal intubation has benefits, physicians need to carefully balance the advantages of averting intubation with the risk of treatment failure, which leads to delayed intubation and worse clinical outcomes [64]. In this post hoc analysis of the HENIVOT trial, helmet NIV was not associated with poorer respiratory outcomes compared to high-flow, ruling out the possibility of long-term detrimental effects related to P-SILI caused by helmet NIV. This is likely due to the strict monitoring of patients and pre-specified criteria for endotracheal intubation, which limited intubation delay, with a median time between enrollment and intubation of 29 h [IQR 8–71] for the helmet group and 21 h [IQR 4–65] for high-flow group [13]. In addition, despite prolonged treatments (48 h continuously in 91% of patients) and lower comfort with helmet NIV compared to high-flow [13], no difference was observed in terms of psychological effects. Given comparable physical and psychological medium-term effects, and the possible beneficial effect on endotracheal intubation, helmet NIV as applied in the HENIVOT trial might be an effective and safe strategy in acute hypoxemic failure.

Our study has several strengths: a systematic approach with a well-defined follow-up protocol, a multicenter involvement, a comprehensive assessment of physical and mental health status, a small number of patients lost to follow-up and few missing data.

Our study has some limitations. First, despite a high level of adherence to follow-up (71 participants out of 80 patients alive at 6 months), the sample might be underpowered to detect whether the need for endotracheal intubation and invasive mechanical ventilation mediates the effect of applied interventions on analyzed outcomes. Second, many indices showed a difference between the two groups, although not significant, and we cannot exclude that a larger sample size might have revealed differences. Finally, the lack of pulmonary function tests before COVID-19 infection did not allow us to discriminate the effect of COVID-19 vs. the effects of P-SILI and ventilator-induced lung injury; however, the randomized design of the investigation should have made the two cohorts comparable.

## Conclusion

In patients with COVID-19 requiring noninvasive respiratory support due to acute hypoxemic respiratory failure, the use of helmet noninvasive ventilation appears as effective and safe as high-flow oxygen, in terms of 6-month functional status and quality of life. Need for invasive mechanical ventilation after noninvasive support is strongly associated with worse quality of life, physical and psychological status at 6 months. These data indicate that helmet NIV, as applied in the HENIVOT trial, can be safely used in hypoxemic patients.

## Supplementary Information

**Additional file 1.**

**Additional file 2.**

**Additional file 3.**

**Additional file 4.**

## Data Availability

The data sets used and/or analyzed during the current study are available from the corresponding author on reasonable request.

## Abbreviations

COVID-19: Coronavirus Disease appeared in 2019
DLCO: Diffusion lung capacity for carbon monoxide
EQ-5D-5L: The EuroQoL five dimensions five levels test
EQ VAS: The EuroQol Visual Analogue Scale
HENIVOT: Randomized clinical trial entitled “Effect of helmet noninvasive ventilation vs. high-flow nasal oxygen on days free of respiratory support in patients with COVID-19 moderate-to-severe hypoxemic respiratory failure”
mMRC: Modified British Medical Research Council
NIV: Non-invasive ventilation
PCL-5: Post Traumatic Stress Disorder Checklist for the Diagnostic and Statistical Manual of mental disorder (DMS-5)
SF 36: Medical Outcomes Study 36 Items Short Form
30SCST: 30-Seconds chairs stand test
6MWT: Six minutes walking test
