# Supplementary figures and images for: Long-term outcome of COVID-19 patients treated with helmet noninvasive ventilation vs. high-flow nasal oxygen: a randomized trial
Source: J Intensive Care. 2023 May 19;11:21. doi: 10.1186/s40560-023-00669-0 (PMC10195662; doi:10.1186/s40560-023-00669-0)

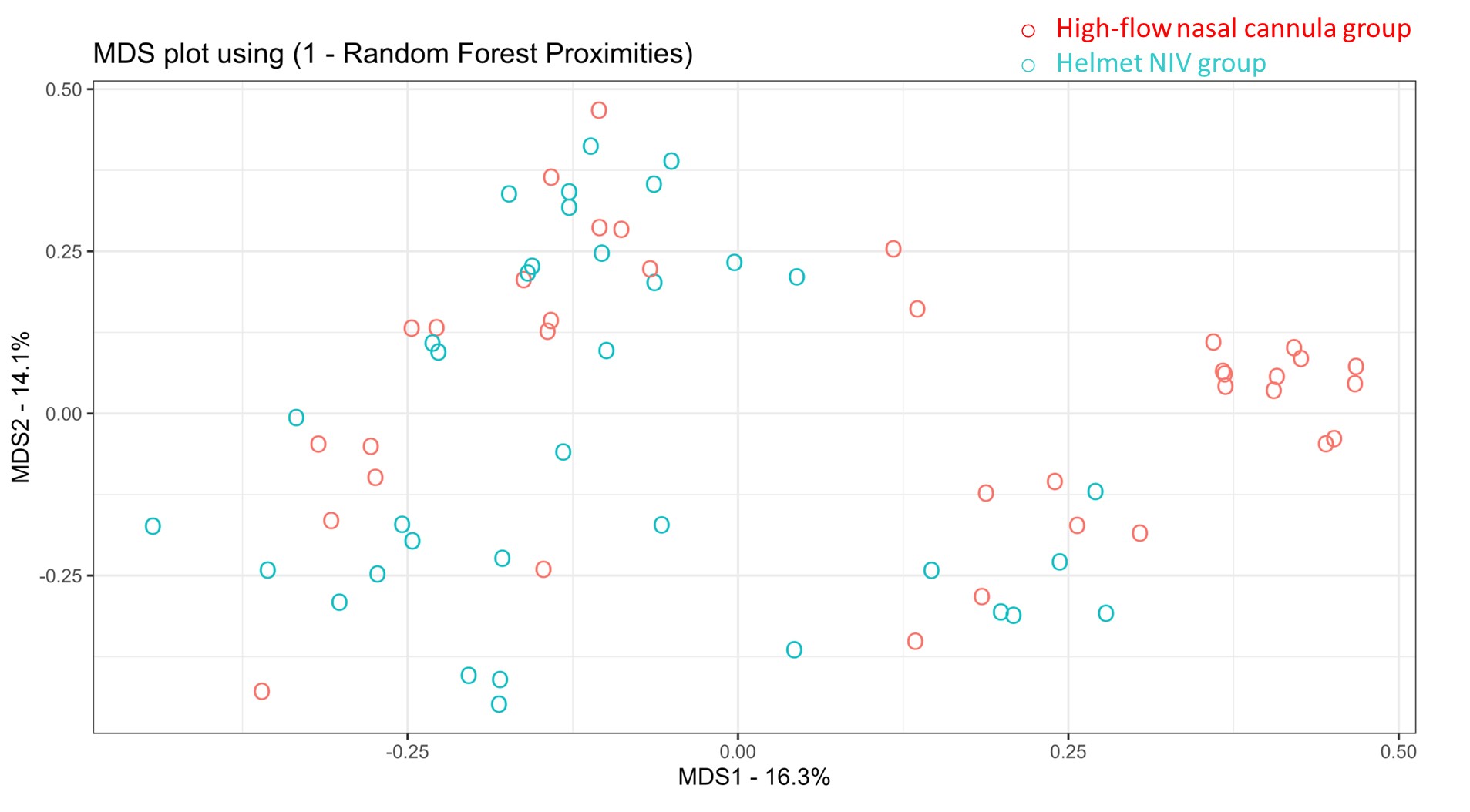

Supplement: Supplementary file 1 — Additional file 1. [file 40560_2023_669_MOESM1_ESM.jpg]

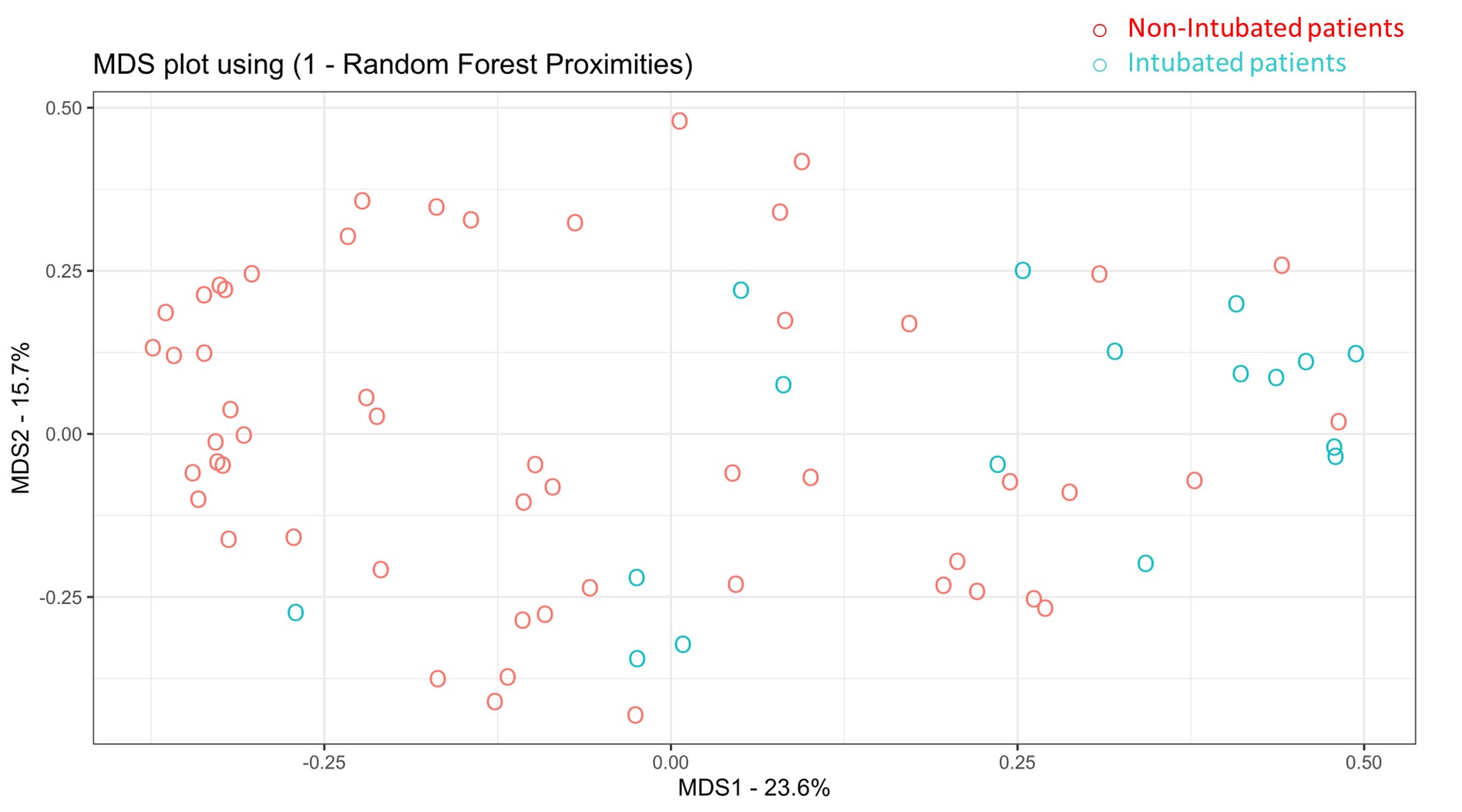

Supplement: Supplementary file 2 — Additional file 2. [file 40560_2023_669_MOESM2_ESM.jpg]
