# Supplementary material for: Long-term outcome of COVID-19 patients treated with helmet noninvasive ventilation vs. high-flow nasal oxygen: a randomized trial
Source: J Intensive Care. 2023 May 19;11:21. doi: 10.1186/s40560-023-00669-0 (PMC10195662; doi:10.1186/s40560-023-00669-0)
Supplement: Supplementary file 3 — Additional file 3. [file 40560_2023_669_MOESM3_ESM.docx]

| **Table S1. Clinical and laboratory assessment at six months, according to study group. *** | | | | |
| --- | --- | --- | --- | --- |
| **Vital signs** | **Helmet Noninvasive Ventilation**  **(n=31)** | **High-flow nasal oxygen**  **(n=31)** | **Absolute or mean difference (95% CI)** | **P value** |
| Spo2 - % | 97 [97-98] | 97 [96-98] | -0.42 (-1.099 to 0.25) | 0.24 |
| Heart rate -beats per minutes | 73 [67-80] | 76 [65-85] | -2.00 (-7.98 to 3.97) | 0.49 |
| Systolic blood pressure - mmHg | 130 [120-140] | 130 [120-140] | 3.10 (-3.57 to 9.78) | 0.35 |
| Diastolic blood pressure -mmHg | 80 [75-80] | 80 [70-80] | 4.48 (-2.17 to 11.13) | 0.33 |
| Respiratory rate – breaths per minute | 28 [24 - 30] | 26 [22 - 31] | -1 (-2 to 4) | 0.52 |
| Dyspnea at enrollment^a^ | 36 [20 - 70] | 30 [20 - 65] | 0 (-1 to 2) | 0.57 |
| Device-related discomfort at enrollment^a^ | 0 [0 - 43] | 0 [0 - 12] | 0 (-7 to 15) | 0.78 |
|  | | | | |
| **Arterial Blood Gas** | **Helmet Noninvasive Ventilation**  **(n=31)** | **High-flow nasal oxygen**  **(n=34)** |  |  |
| pH | 7.42 [7.41-7.44] | 7.41 [7.39-7.42] | -0.01 (-0.03 to -0.0001) | 0.02 |
| PaO_2_ -mmHg | 84 [78-91] | 87 [80-94] | 3.09 (-2.23 to 8.42) | 0.25 |
| PaCO_2_ - mmHg | 38 [36.3-41.9] | 39 [37-42] | 0.24 (-1.66 to 2.14) | 0.46 |
| HCO_3_^-^ - mmol/L | 25 [24.4-26.8] | 25 [24-27] | -0.91 (-1.87 to -0.002) | 0.15 |
| P(A-a)O_2_ -mmHg | 19 [14-24] | 15 [11-21] | -3.41 (-7.49 to 0.67) | 0.13 |
| PaO_2_/FiO_2_ | 400 [372-435] | 417 [395-446] | 17.98 (-8.26 to 44.22) | 0.18 |
|  | | | | |
| **Laboratory variables** | **Helmet Noninvasive Ventilation**  **(n=29)** | **High-flow nasal oxygen**  **(n=29)** |  |  |
| Hemoglobin – g/dl | 14.5 [13.65-15.55] | 14.7 [13.8-15.8] | -0.21 (-1.33 to 0.92) | 0.76 |
| Platelets -10^9^/L | 226 [172.5-258] | 211 [179-245] | -4.38 (-34.06 to 25.30) | 0.52 |
| White blood cells count – 10^9^/L | 6 [5.26-7.49] | 6.8 [6.05-7.82] | 0.64 (-0.11 to 1.40) | 0.06 |
| Creatinine - mg/dl | 0.83 [0.67-1.07] | 0.85 [0.66-1.04] | 0.04 (-0.11 to 0.19) | 0.85 |
| Blood Urea Nitrogen - mg/dl | 20 [17-25] | 18 [16-26] | -0.90 (-3.84 to 2.05) | 0.51 |
| Estimated Glomerular Filtration Rate – mL/min/1.73m^2^ | 89 [71-98] | 88 [79-102] | 5.25 (-6.89 to 17.39) | 0.47 |
| Glutamate-oxaloacetate transaminase - mg/dl | 19 [15-23] | 18 [15-24] | -0.99 (-6.03 to 4.05) | 0.99 |
| Glutamate-pyruvate transaminase- mg/dl | 21 [14-33] | 20 [11-29] | -5.79 (-13.90 to 2.31) | 0.36 |
| Total bilirubin - mg/dl | 0.6 [0.5-1] | 0.7 [0.6-1] | 0.09 (-0.10 to 0.28) | 0.28 |
| ^* Values are displayed as medians [interquartile range], if not otherwise specified.^  ^a Dyspnea and Discomfort were assessed through visual analogue scales adapted for intensive care unit patients ranging from 0 to 10.^ | | | | |

| **Table S2. Medical assessment at six months, according to study group. *** | | | | |
| --- | --- | --- | --- | --- |
| **Pulmonary performance test^+^** | **Helmet Noninvasive Ventilation**  **(n=30)** | **High-flow nasal oxygen**  **(n=30)** | **Absolute or mean difference (95% CI)** | **P value** |
| Forced vital capacity – litres | 3.80 [2.91-4.21] | 3.75 [2.70-4.40] | 0.03 (-0.46 to 0.53) | 0.84 |
| Forced expiratory volume in one second - litres | 2.9 [2.19-3.47] | 3.14 [2.21-3.67] | 0.10 (-0.32 to 0.51) | 0.62 |
| Total lung capacity – litres | 5.79 [4.51-6.44] | 5.67 [4.13-6.32] | -0.21 (-0.88 to 0.46) | 0.58 |
| Diffusing capacity of the lung for carbon monoxide – ml/min/mmHg | 19 [16-23] | 22 [16-24] | 1.49 (-1.4 to 4.37) | 0.48 |
| Alveolar ventilation - litres | 5.41 [4.32-5.99] | 5.45 [4.29-5.86] | 0.11 (-0.50 to 0.72) | 0.95 |
| Residual volume - litres | 1.92 [1.52-2.32] | 1.81 [1.58-2.09] | -0.14 (-0.4 to 0.12) | 0.44 |
|  | | | | |
| **30 second chair stand test** | **Helmet Noninvasive Ventilation**  **(n=26)** | **High-flow nasal oxygen**  **(n=24)** |  |  |
| Number of cycles – N (%) | 23 [20-29] | 23 [19-25] | -1.19 (-4.86 to 2.49) | 0.65 |
| SpO_2_ nadir - % | 95 [93-96] | 95 [93-96] | 0.59 (-0.79 to 1.97) | 0.42 |
| Higher heart rate- beats per minutes | 100 [86-114] | 100 [87-112] | 0.05 (-8.83 to 8.94) | 0.95 |
| SpO_2_ at the end of the test - % | 95 [93-96] | 95 [94-96] | 0.16 (-1.20 to 1.52) | 0.96 |
| Heart rate at the end of the test- beats per minutes | 96 [80-112] | 100 [87-112] | 3.31 (-6.37 to 12.98) | 0.54 |
| SpO_2_ after 4 minutes of rest - % | 97 [97-98] | 97 [96-98] | -0.16 (-0.99 to 0.67) | 0.59 |
| Heart rate after 4 minutes of rest- beats per minutes | 78 [69-90] | 78 [74-80] | -1.03 (-7.42 to 5.36) | 0.98 |
| BORG scale for dyspnea (range 0-10) | 4 [2-5] | 5 [2-7] | 0.41 (-0.93 to 1.76) | 0.50 |
| Test interruption – N (%) | 0 (0) | 0 (0) | 0 (-14 to 13) | - |
|  | | | | |
| **Quality of life assessment: EQ-5D-5L^+^** | **Helmet Noninvasive Ventilation**  **(n=35)** | **High-flow nasal oxygen**  **(n=36)** |  |  |
| Mobility (range 1-5) | 1 [1-2] | 1 [1-2] | 0.29 (-0.12 to 0.71) | 0.26 |
| Personal care (range 1-5) | 1 [1-1] | 1 [1-1] | 0.19 (-0.20 to 0.57) | 0.64 |
| Usual activities (range 1-5) | 1 [1-2] | 2 [1-2] | 0.35 (-0.10 to 0.79) | 0.10 |
| Pain or discomfort (range 1-5) | 2 [1-2] | 2 [1-3] | 0.20 (-0.22 to 0.62) | 0.48 |
| Anxiety or depression (range 1-5) | 1 [1-2] | 1 [1-2] | 0.10 (-0.35 to 0.54) | 0.71 |
| ^* Values are displayed as medians [interquartile range].^  ^+ For the results expressed as % of predicted value or as no. of impaired patients (with performance lower than 80% of predicted see Table 2).^ | | | | |

| **Table S3. Characteristics of patients, according to previous intubation. *** | | | |
| --- | --- | --- | --- |
| **Characteristic** | **Non-intubated patients**  **(n=54)** | **Intubated patients**  **(n=17)** | **P value** |
| Age – years | 64 [54-72] | 59 [53-67] | 0.35 |
| Female sex – N (%) | 12 (22) | 1 (6) | 0.17 |
| Body Mass index§ | 27.8 [25.4-30.5] | 27.8 [25.4-31.5] | 0.95 |
| Most relevant comorbidities | | | |
| Hypertension – N (%) | 25 (46) | 9 (53) | 0.78 |
| Type 2 diabetes mellitus – N (%) | 10 (19) | 4 (24) | 0.73 |
| Smoking – N (%) | 20 (37) | 8 (47) | 0.57 |
| Immunocompromised state – N (%) | 5 (9) | 1 (6) | >0.99 |
| Recent chemotherapy – N (%) | 1 (2) | 0 (0) | - |
| Human Immunodeficiency Virus – N (%) | 1(2) | 0 (0) | - |
| Immunosuppressive therapy-renal transplant – N (%) | 1 (2) | 0 (0) | - |
| Acute myeloid leukemia – N (%) | 0 (0) | 1 (6) | - |
| Ulcerative colitis-immunosuppressive therapy – N (%) | 2 (4) | 0 (0) | - |
| History of cancer – N (%) | 3 (6) | 1 (6) | >0.99 |
| Neurological conditions – N (%) | 0 (0) | 0 (0) | - |
| Randomized to helmet during hospitalization – N (%) | 32 (59) | 3 (18) | 0.005 |
| ^* Values are displayed as medians [interquartile range], if not otherwise specified. § The body-mass index is the weight in kilograms divided by the square of the height in meters.^ | | | |

| **Table S4. Medical assessment at six months. according to previous intubation. *** | | | | |
| --- | --- | --- | --- | --- |
|  | | | | |
| **Vital signs** | **Non-intubated patients**  **( N=47)** | **Intubated patients**  **(N=15)** | **Absolute or mean difference (95% CI)** | **P value** |
| Spo2 - % | 97 [96-98] | 97 [96-97] | 0.49 (-0.31 to 1.21) | 0.15 |
| Heart rate - beats per minutes | 76 [67-83] | 74 [65-81] | 2.35 (-4.43 to 9.13) | 0.49 |
| Systolic blood pressure - mmHg | 130 [120-140] | 128 [115-140] | 4.01 (-3.77 to 11.79) | 0.31 |
| Diastolic blood pressure -mmHg | 80 [70-80] | 80 [70-80] | 1.09 (-6.80 to 8.98) | 0.20 |
|  | | | | |
| **Arterial Blood Gas** | **Non-intubated patients**  **( N=48)** | **Intubated patients**  **(N=17)** |  |  |
| pH | 7.42 [7.41-7.43] | 7.40 [7.39-7.42] | 0.01 (0.00 to 0.03) | 0.03 |
| PaO_2_ -mmHg | 87 [81-93] | 83 [78-91] | 2.97 (-3.10 to 9.04) | 0.30 |
| PaCO_2_ - mmHg | 38 [37-41] | 39 [37-43] | -1.47 (-3.59 to 0.66) | 0.21 |
| HCO_3_^-^ - mmol/L | 25 [24-27] | 25 [23-26] | 0.62 (-0.42 to 1.67) | 0.31 |
| P(A-a)O_2_ -mmHg | 18 [11-22] | 19 [12-26] | -1.86 (-6.69 to 2.98) | 0.52 |
| PaO_2_/FiO_2_ | 415 [388-443] | 409 [379-432] | 11.73 (-18.39 to 41.84) | 0.48 |
|  | | | | |
| **Blood tests** | **Non-intubated patients**  **( N=44)** | **Intubated patients**  **(N=14)** |  |  |
| Hemoglobin – g/dl | 14.6 [13.7-15.6] | 14.5 [13.0-15.6] | -0.15 (-1.46 to 1.16) | 0.79 |
| Platelets -10^9^/L | 214 [188-252] | 220 [165-250] | 18.88 (-15.46 to 53.22) | 0.50 |
| White blood cells count – 10^9^/L | 6.19 [5.64-7.76] | 7.07 [6.08-7.93] | -0.61 (-1.50 to 0.27) | 0.17 |
| Creatinine - mg/dl | 0.78 [0.65-1.01] | 1.00 [0.79-1.32] | -0.18 (-0.36 to -0.01) | 0.06 |
| Blood Urea Nitrogen - mg/dl | 18 [16-25] | 23 [15-27] | -2.37 (-5.76 to 1.02) | 0.17 |
| Estimated Glomerular Filtration Rate – mL/min/1.73m^2^ | 92 [78-101] | 80 [58-91] | 14.44 (0.80 to 28.09) | 0.04 |
| Glutamate-oxaloacetate transaminase - mg/dl | 19 [16-25] | 18 [13-24] | 1.56 (-4.57 to 7.69) | 0.38 |
| Glutamate-pyruvate transaminase - mg/dl | 21 [15-31] | 19 [8-32] | 4.61 (-4.95 to 14.18) | 0.22 |
| Total bilirubin - mg/dl | 0.6 [0.5-0.97] | 0.7 [0.6-1.1] | -0.07 (-0.29 to 0.14) | 0.24 |
|  | | | | |
| **Symptoms** | **Non-intubated patients**  **(N=47)** | **Intubated patients**  **(N=17)** | **Absolute or mean difference (95% CI)** | **P value** |
| Dyspnoea – N (%) | 31 (66) | 12 (71) | -5 (-26 to 22) | >0.99 |
| mMRC scale (range 0-4) | 2 [2-2] | 2 [2-3] | -0.31 (-0.69 to 0.08) | 0.12 |
| Dry cough – N (%) | 2 (4) | 3 (18) | -14 (-37 to 2) | 0.11 |
| Sore throat – N (%) | 0 (0) | 2 (12) | -12 (-34 to 0) | 0.07 |
| Productive cough – N (%) | 0 (0) | 0 (0) | 0 (-18 to 8) | - |
| Rhinitis – N (%) | 1 (2) | 2 (12) | -10 (-32 to 3) | 0.17 |
| Smell disorder – N (%) | 3 (6) | 1 (6) | 0 (-21 to 12) | >0.99 |
| Decreased visual acuity – N (%) | 4 (9) | 3 (18) | -9 (-33 to 7) | 0.37 |
| Conjunctival hyperaemia – N (%) | 0 (0) | 0 (0) | 0 (-18 to 8) | - |
| Taste disorder – N (%) | 3 (6) | 1 (6) | 1 (-21 to 12) | >0.99 |
| Inappetence – N (%) | 2 (4) | 1 (6) | -2 (-23 to 9) | >0.99 |
| Diarrhoea – N (%) | 3 (6) | 1 (6) | 1 (-21 to 12) | >0.99 |
| Myalgia – N (%) | 12 (26) | 9 (53) | -27 (-51 to 1) | 0.07 |
| Chest pain – N (%) | 3 (6) | 2 (12) | -5 (-28 to 8) | 0.60 |
| Sicca syndrome – N (%) | 1 (2) | 1 (6) | -4 (-25 to 6) | 0.46 |
| Raynaud syndrome – N (%) | 0 (0) | 0 (0) | 0 (-18 to 8) | - |
| Skin lesion – N (%) | 0 (0) | 0 (0) | 0 (-18 to 8) | - |
| Syncope – N (%) | 0 (0) | 0 (0) | 0 (-18 to 8) | - |
| Dizziness – N (%) | 0 (0) | 1 (6) | -6 (-27 to 3) | 0.27 |
| Headache – N (%) | 0 (0) | 0 (0) | 0 (-18 to 8) | - |
|  | | | | |
| **Pulmonary function test** | **Non-intubated patients**  **(N=45)** | **Intubated patients**  **(N=15)** |  |  |
| Forced vital capacity - litres | 3.85 [2.95-4.46] | 3.25 [2.67-4.21] | 0.29 (0.28 to 0.86) | 0.35 |
| Forced expiratory volume in one second - litres | 3.03 [2.16-3.69] | 2.70 [2.27-3.44] | 0.11 (-0.36 to 0.59) | 0.74 |
| Forced expiratory volume in one second % of predicted | 96 [88-106] | 85 [76-99] | 10.9 (2.11 to 19.69) | 0.05 |
| Forced expiratory volume in one second <80% of predicted – N (%) | 5 (11) | 5 (33) | -22 (-48 to 1) | 0.10 |
| Forced expiratory volume in one second /  Forced vital capacity ratio | 0.81 [0.77-0.84] | 0.85 [0.81-0.88] | -4 (-8 to 0) | 0.03 |
| Forced expiratory volume in one second /  Forced vital capacity ratio % of predicted | 103 [100-107] | 108 [101-115] | -3.82 (-9.45 to 1.80) | 0.07 |
| Forced expiratory volume in one second /  Forced vital capacity ratio <80 % of predicted – N (%) | 16 (35) | 2(13) | 21 (-6 to 39) | 0.19 |
| Maximal (mid-) expiratory flow 25-75 – litres per second | 1.13 [0.98-1.33] | 1.22 [0.85-1.64] | -0.10 (-0.30 to 0.09) | 0.52 |
| Total lung capacity – litres | 6.01 [4.51-6.56] | 5.18 [4.06-6.15] | 0.53 (-0.23 to 1.30) | 0.15 |
| Diffusing capacity of the lung for carbon monoxide – ml/min/mmHg | 20.27 [17.39-24.87] | 15.54 [13.25-21.84] | 3.46 (0.14 to 6.78) | 0.03 |
| Diffusing capacity of the lung for carbon monoxide / alveolar ventilation ratio | 0.94 [0.86-1.05] | 0.96 [0.84-1.01] | 0.02 (-0.09 to 0.13) | 0.81 |
| Alveolar ventilation - litres | 5.49 [4.41-6.21] | 4.78 [3.80-5.60] | 0.72 (0.02 to 1.42) | 0.03 |
| Diffusing capacity of the lung for carbon monoxide / alveolar ventilation ratio | 0.94 [0.86-1.05] | 0.96 [0.84-1.01] | 0.02 (-0.09 to 0.13) | 0.81 |
| Residual volume - litres | 1.83 [1.57-2.32] | 1.67 [1.30-1.94] | 0.30 (0.01 to 0.60) | 0.05 |
| Residual volume / total lung capacity ratio | 34.57 [29.42-39.13] | 33.00 [30.72-34.39] | 2.08 (-1.80 to 5.95) | 0.20 |
|  | | | | |
| **Six minutes walking test** | **Non-intubated patients**  **( N=46)** | **Intubated patients**  **(N=14)** | **Absolute or mean difference (95% CI)** | **P value** |
| SpO2 nadir during test - % | 94 [91-95] | 94 [90-95] | 0.01 (-0.009 to 0.03) | 0.74 |
| Recovery time to return SpO2 to basal value - minutes | 1 [1-2] | 2 [1-2] | -0.18 (-0.80 to 0.43) | 0.59 |
| Maximum heart rate - beats per minutes | 107 [99-114] | 101 [98-113] | 1.23 (-6.88 to 9.34) | 0.44 |
| Recovery time to return heart rate to basal value – minutes | 2 [2-3] | 2 [1-3] | 0.23 (-0.35 to 0.81) | 0.51 |
| BORG scale for dyspnea during test (range 0-10) | 3 [2-5] | 3 [3-6] | -0.39 ( -1.73 to 1.06) | 0.49 |
| Interruption of test – N (%) | 1 (2) | 0 (0) | 2 (-19 to 12) | >0.99 |
|  | | | | |
| **30 second chair stand test** | **Non-intubated patients**  **( N=39)** | **Intubated patients**  **(N=11)** |  |  |
| Number of cycles – N (%) | 24 [20-27] | 20 [16-25] | 2.94 (-1.43 to 7.31) | 0.15 |
| SpO_2_ nadir - % | 95 [94-96] | 95 [92-96] | 0.75 (-0.92 to 2.41) | 0.53 |
| Higher heart rate- beats per minutes | 103 [86-113] | 100 [86-107] | 4.19 (-6.46 to 14.84) | 0.43 |
| SpO_2_ at the end of the test - % | 96 [94-97] | 95 [92-96] | 1.58 (0.01 to 3.16) | 0.09 |
| Heart rate at the end of the test- beats per minutes | 97 [80-113] | 99 [81-107] | 0.59 (-11.13 to 12.32) | 0.87 |
| SpO_2_ after 4 minutes of rest - % | 97 [97-98] | 97 [96-97] | 0.30 (-0.70 to 1.30) | 0.39 |
| Heart rate after 4 minutes of rest- beats per minutes | 80 [70-87] | 76 [65-80] | 4.69 (-2.90 to 12.29) | 0.28 |
| BORG scale for dyspnea (range 0-10) | 4 [2-6] | 4 [3-6] | -0.38 (-2.00 to 1.25) | 0.56 |
| Test interruption – N (%) | 0 (0) | 0 (0) | 0 (-26 to 9) | - |
|  | | | | |
| **EQ-5D-5L** | **Non-intubated patients**  **( N=54)** | **Intubated patients**  **(N=17)** |  |  |
| Mobility (range 1-5) | 1 [1-2] | 2 [1-3] | -0.67 (-1.13 to -0.21) | 0.008 |
| Personal care (range 1-5) | 1 [1-1] | 1 [1-3] | -0.35 (-0.80 to 0.10) | 0.10 |
| Usual activities (range 1-5) | 1 [1-2] | 2 [1-3] | -0.56 (-1.08 to -0.05) | 0.01 |
| Pain discomfort (range 1-5) | 2 [1-2] | 2 [2-3] | -0.51 (-0.99 to -0.03) | 0.04 |
| Anxiety depression (range 1-5) | 1 [1-2] | 2 [1-3] | -0.19 (-0.71 to 0.33) | 0.63 |
| ^* Values are displayed as medians [interquartile range].^ | | | | |

| **Table S5. Correlation between time before endotracheal intubation in hours and functional outcome at six months.** | | |
| --- | --- | --- |
|  | **Hours of respiratory support before endotracheal intubation** | |
|  | **r** | **p** |
| **Pulmonary function test** |  |  |
| Residual volume - % of predicted | 0.61 | 0.016 |
| Residual volume and total lung capacity ratio - % of predicted | 0.52 | 0.05 |
| **30-second chair stand test** |  |  |
| Nadir SpO2 during 30-second chair stand test - % | 0.71 | 0.013 |
| Time to recover SpO2 after 30-second chair stand test - seconds | 0.61 | 0.05 |
| Time to recover heart rate after 30-second chair stand test -seconds | 0.66 | 0.03 |
