# Supplementary material for: Long-term outcome of COVID-19 patients treated with helmet noninvasive ventilation vs. high-flow nasal oxygen: a randomized trial
Source: J Intensive Care. 2023 May 19;11:21. doi: 10.1186/s40560-023-00669-0 (PMC10195662; doi:10.1186/s40560-023-00669-0)
Supplement: Supplementary file 4 — Additional file 4. [file 40560_2023_669_MOESM4_ESM.docx]

**Appendix**

**Table of contents:**

1. Additional figure legend
2. Modified Medical Research Council (mMRC) Dyspnea Scale
3. The 36-Item Short Form Survey (SF 36)
4. The Post Traumatic Stress Disorder Checklist for the Diagnostic and Statistical Manual of mental disorder (DMS-5) (PCL 5)
5. The 5-level EQ-5D version (EQ-5D-5L)
6. Questionnaire on COVID-19 related symptoms at six months
7. **Additional figure legend**

**Additional** figure S1

In the figure, the Random Forest proximity plot is presented. This graphical representation displays the relationships among the observations in the dataset based on their proximity values derived from the Random Forest analysis. The plot visually reflects the similarity between pairs of observations, measured by the frequency with which they end up in the same terminal nodes of the trees in the Random Forest model.

In panel **a)**, the proximity plot shows the clustering of patients treated with high-flow nasal cannula (represented by red dots) or helmet NIV (represented by green dots), and the selected variables: EQ-VAS, diffusing lung capacity for carbon monoxide percentage, total lung capacity, Tiffeneau index, and occurrence of arthralgia at follow-up. SAPS II and age were added to the model as confounding factors. Each point represents an individual observation. Observations on the left side of the graph indicate patients who performed better, while patients on the right side of the graph performed worse. This model had higher uncertainties in its predictive power; however, three populations are identifiable: a general population with good functional outcomes at follow-up, populated with patients from both groups (blue circle), a population with worse functional outcomes at follow-up, populated with patients from both groups (red circle), and a small cluster of only high-flow nasal cannula patients with worse functional outcomes at follow-up (green circle).

In panel **b)**, the proximity plot shows clustering between intubated and non-intubated patients and the selected variables (the same as in panel **a)**). This model had less uncertainty in its predictive power (MSD 19.72%), as visually appreciated by the asymmetry of the clustering. Patients who received endotracheal intubation are more likely to be on the right side of the graph, while those who did not receive endotracheal intubation are on the left side. However, the small sample size (mainly the small number of intubated patients) precludes clear clustering in this visualization.

1. **Modified Medical Research Council (mMRC) Dyspnea Scale**

The mMRC scale is a clinical tool to stratify dyspnea (the score going from 0 to 4) according to

breathlessness during different intensity activity.

| **Grade** | **Description of breathlessness** |
| --- | --- |
| 0 | I only get breathless with strenuous exercise |
| 1 | I get short of breath when hurrying on level ground or walking up a slight hill |
| 2 | On level ground, I walk slower than people of the same age because of breathlessness, or have to stop for breath when walking at my own pace |
| 3 | I stop for breath after walking about 100 yards or after a few minutes on level ground |
| 4 | I am too breathless to leave the house or I am breathless when dressing |

References

1. FLETCHER CM, ELMES PC, FAIRBAIRN AS, WOOD CH. The significance of respiratory symptoms and the diagnosis of chronic bronchitis in a working population. *Br Med J*. 1959;2(5147):257-266. doi:10.1136/bmj.2.5147.257
2. Munari AB, Gulart AA, Dos Santos K, Venâncio RS, Karloh M, Mayer AF. Modified Medical Research Council Dyspnea Scale in GOLD Classification Better Reflects Physical Activities of Daily Living. *Respir Care*. 2018;63(1):77-85. doi:10.4187/respcare.05636
3. **The 36-Item Short Form Survey (SF 36)**

The 36-Item Short Form Health Survey (SF-36) has been developed as part of the Medical Outcomes Study (MOS), a multi-year study to explain variations in patient outcomes^1^.

The SF-36 is a 36-item patient-report questionnaire on quality of life; it consists of eight different

scale summarized by two derived score, regarding mental health and physical health. The higher

score represents a better subjective health experience. It was designed for use in clinical practice and research, health policy evaluations and general population surveys. It relies on patient’s self-reporting measures.

The Italian version of the MOS 36-Item Short Form Health Survey (SF-36) has been validated in the context of the International Quality Of Life Assessment (IQOLA) project in 1991^2^.
We agree and accepted terms and conditions for using the IQOLA SF-36 Italian version.

References

1. Ware JE Jr, Sherbourne CD. The MOS 36-item short-form health survey (SF-36). I. Conceptual framework and item selection. *Med Care*. 1992;30(6):473-483.
2. Apolone G, Mosconi P. The Italian SF-36 Health Survey: translation, validation and norming. *J Clin Epidemiol*. 1998;51(11):1025-1036. doi:10.1016/s0895-4356(98)00094-8

36-Item Short Form**:**

**Choose one option for each questionnaire item.**

Inizio modulo

1. In general, would you say your health is:

- 1 - Excellent
- 2 - Very good
- 3 - Good
- 4 - Fair
- 5 - Poor

2. **Compared to one year ago**, how would you rate your health in general **now**?

- 1 - Much better now than one year ago
- 2 - Somewhat better now than one year ago
- 3 - About the same
- 4 - Somewhat worse now than one year ago
- 5 - Much worse now than one year ago

The following items are about activities you might do during a typical day. Does **your health now limit you** in these activities? If so, how much?

|  | Yes, limited a lot | Yes, limited a little | No, not limited at all |
| --- | --- | --- | --- |
| 3. **Vigorous activities**, such as running, lifting heavy objects, participating in strenuous sports | 1 | 2 | 3 |
| 4. **Moderate activities**, such as moving a table, pushing a vacuum cleaner, bowling, or playing golf | 1 | 2 | 3 |
| 5. Lifting or carrying groceries | 1 | 2 | 3 |
| 6. Climbing **several** flights of stairs | 1 | 2 | 3 |
| 7. Climbing **one** flight of stairs | 1 | 2 | 3 |
| 8. Bending, kneeling, or stooping | 1 | 2 | 3 |
| 9. Walking **more than a mile** | 1 | 2 | 3 |
| 10. Walking **several blocks** | 1 | 2 | 3 |
| 11. Walking **one block** | 1 | 2 | 3 |
| 12. Bathing or dressing yourself | 1 | 2 | 3 |

During the **past 4 weeks**, have you had any of the following problems with your work or other regular daily activities **as a result of your physical health**?

|  | Yes | No |
| --- | --- | --- |
| 13. Cut down the **amount of time** you spent on work or other activities | 1 | 2 |
| 14. **Accomplished less** than you would like | 1 | 2 |
| 15. Were limited in the **kind** of work or other activities | 1 | 2 |
| 16. Had **difficulty** performing the work or other activities (for example, it took extra effort) | 1 | 2 |

During the **past 4 weeks**, have you had any of the following problems with your work or other regular daily activities **as a result of any emotional problems** (such as feeling depressed or anxious)?

|  | Yes | No |
| --- | --- | --- |
| 17. Cut down the **amount of time** you spent on work or other activities | 1 | 2 |
| 18. **Accomplished less** than you would like | 1 | 2 |
| 19. Didn't do work or other activities as **carefully** as usual | 1 | 2 |

20. During the **past 4 weeks**, to what extent has your physical health or emotional problems interfered with your normal social activities with family, friends, neighbors, or groups?

- 1 - Not at all
- 2 - Slightly
- 3 - Moderately
- 4 - Quite a bit
- 5 - Extremely

21. How much **bodily** pain have you had during the **past 4 weeks**?

- 1 - None
- 2 - Very mild
- 3 - Mild
- 4 - Moderate
- 5 - Severe
- 6 - Very severe

22. During the **past 4 weeks**, how much did **pain** interfere with your normal work (including both work outside the home and housework)?

- 1 - Not at all
- 2 - A little bit
- 3 - Moderately
- 4 - Quite a bit
- 5 - Extremely

These questions are about how you feel and how things have been with you **during the past 4 weeks**. For each question, please give the one answer that comes closest to the way you have been feeling.

How much of the time during the **past 4 weeks**...

|  | All of the time | Most of the time | A good bit of the time | Some of the time | A little of the time | None of the time |
| --- | --- | --- | --- | --- | --- | --- |
| 23. Did you feel full of pep? | 1 | 2 | 3 | 4 | 5 | 6 |
| 24. Have you been a very nervous person? | 1 | 2 | 3 | 4 | 5 | 6 |
| 25. Have you felt so down in the dumps that nothing could cheer you up? | 1 | 2 | 3 | 4 | 5 | 6 |
| 26. Have you felt calm and peaceful? | 1 | 2 | 3 | 4 | 5 | 6 |
| 27. Did you have a lot of energy? | 1 | 2 | 3 | 4 | 5 | 6 |
| 28. Have you felt downhearted and blue? | 1 | 2 | 3 | 4 | 5 | 6 |
| 29. Did you feel worn out? | 1 | 2 | 3 | 4 | 5 | 6 |
| 30. Have you been a happy person? | 1 | 2 | 3 | 4 | 5 | 6 |
| 31. Did you feel tired? | 1 | 2 | 3 | 4 | 5 | 6 |

32. During the **past 4 weeks**, how much of the time has **your physical health or emotional problems** interfered with your social activities (like visiting with friends, relatives, etc.)?

- 1 - All of the time
- 2 - Most of the time
- 3 - Some of the time
- 4 - A little of the time
- 5 - None of the time

How TRUE or FALSE is **each** of the following statements for you.

|  | Definitely true | Mostly true | Don't know | Mostly false | Definitely false |
| --- | --- | --- | --- | --- | --- |
| 33. I seem to get sick a little easier than other people | 1 | 2 | 3 | 4 | 5 |
| 34. I am as healthy as anybody I know | 1 | 2 | 3 | 4 | 5 |
| 35. I expect my health to get worse | 1 | 2 | 3 | 4 | 5 |
| 36. My health is excellent | 1 | 2 | 3 | 4 | 5 |

Fine modulo

1. **The Post Traumatic Stress Disorder Checklist for the Diagnostic and Statistical Manual of mental disorder (DMS-5) (PCL-5)**

The PCL-5 is a 20-item self-report questionnaire that assesses the 20 symptoms of post-traumatic stress

disorder, according to the Diagnostic and Statistical Manual of mental disorder (DMS-5)^1^.

Respondents rate each item from 0 ("not at all") to 4 ("extremely") to indicate the degree to which they have been bothered by that particular symptom over the past month.

Initial research suggests that a PCL-5 cutoff score between 31-33 is indicative of probable PTSD across samples^2^.

This measure was developed by staff at Veterans Affairs National Center for PTSD and is in the public domain and not copyrighted. In accordance with the American Psychological Association's ethical guidelines, this instrument is intended for use by qualified health professionals and researchers.

References:

1. Weathers, F.W., Litz, B.T., Keane, T.M., Palmieri, P.A., Marx, B.P., & Schnurr, P.P. (2013). The PTSD Checklist for DSM-5 (PCL-5). Scale available from the National Center for PTSD at [www.ptsd.va.gov](https://www.ptsd.va.gov/).
2. Blevins CA, Weathers FW, Davis MT, Witte TK, Domino JL. The Posttraumatic Stress Disorder Checklist for DSM-5 (PCL-5): Development and Initial Psychometric Evaluation. *J Trauma Stress*. 2015;28(6):489-498. doi:10.1002/jts.22059

PCL-5:

| **In the past month, how much were you bothered by:** | **Not at all** | **A little bit** | **Moderately** | **Quite a bit** | **Extremely** |
| --- | --- | --- | --- | --- | --- |
| 1. Repeated, disturbing, and unwanted memories of the stressful experience? | 0 | 1 | 2 | 3 | 4 |
| 2. Repeated, disturbing dreams of the stressful experience? | 0 | 1 | 2 | 3 | 4 |
| 3. Suddenly feeling or acting as if the stressful experience were actually happening again (as if you were actually back there reliving it)? | 0 | 1 | 2 | 3 | 4 |
| 4. Feeling very upset when something reminded you of the stressful experience? | 0 | 1 | 2 | 3 | 4 |
| 5. Having strong physical reactions when something reminded you of the stressful experience (for example, heart pounding, trouble breathing, sweating)? | 0 | 1 | 2 | 3 | 4 |
| 6. Avoiding memories, thoughts, or feelings related to the stressful experience? | 0 | 1 | 2 | 3 | 4 |
| 7. Avoiding external reminders of the stressful experience (for example, people, places, conversations, activities, objects, or situations)? | 0 | 1 | 2 | 3 | 4 |
| 8. Trouble remembering important parts of the stressful experience? | 0 | 1 | 2 | 3 | 4 |
| 9. Having strong negative beliefs about yourself, other people, or the world (for example, having thoughts such as: I am bad, there is something seriously wrong with me, no one can be trusted, the world is completely dangerous)? | 0 | 1 | 2 | 3 | 4 |
| 10. Blaming yourself or someone else for the stressful experience or what happened after it? | 0 | 1 | 2 | 3 | 4 |
| 11. Having strong negative feelings such as fear, horror, anger, guilt, or shame? | 0 | 1 | 2 | 3 | 4 |
| 12. Loss of interest in activities that you used to enjoy? | 0 | 1 | 2 | 3 | 4 |
| 13. Feeling distant or cut off from other people? | 0 | 1 | 2 | 3 | 4 |
| 14. Trouble experiencing positive feelings (for example, being unable to feel happiness or have loving feelings for people close to you)? | 0 | 1 | 2 | 3 | 4 |
| 15. Irritable behavior, angry outbursts, or acting aggressively? | 0 | 1 | 2 | 3 | 4 |
| 16. Taking too many risks or doing things that could cause you harm? | 0 | 1 | 2 | 3 | 4 |
| 17. Being “superalert” or watchful or on guard? | 0 | 1 | 2 | 3 | 4 |
| 18. Feeling jumpy or easily startled? | 0 | 1 | 2 | 3 | 4 |
| 19. Having difficulty concentrating? | 0 | 1 | 2 | 3 | 4 |
| 20. Trouble falling or staying asleep? | 0 | 1 | 2 | 3 | 4 |

1. **The EuroQoL five dimensions five levels (EQ-5D-5L)**

The EuroQoL five dimension five levels (EQ-5D-5L) is an instrument to measure health-related quality of life introduced by the [EuroQol Group](https://euroqol.org/euroqol/) in 2009^1^. The EQ-5D-5L essentially consists of 2 pages: the EQ-5D descriptive system and the EQ visual analogue scale (EQ VAS).

The descriptive system comprises five dimensions: mobility, self-care, usual activities, pain/discomfort and anxiety/depression. Each dimension has 5 levels (ranging from 1 to 5): no problems, slight problems, moderate problems, severe problems and extreme problems. The patient is asked to indicate his/her health state by ticking the box next to the most appropriate statement in each of the five dimensions.

The EQ VAS records the patient’s self-rated health on a vertical visual analogue scale, where the endpoints are labelled ‘The best health you can imagine’ and ‘The worst health you can imagine’. The VAS can be used as a quantitative measure of health outcome that reflect the patient’s own judgement.

According to terms and conditions for using the EuroQoL five dimension five levels, the study has been registered on EuroQoL platform. We obtained the Italian version of the instrument with the related translation certificate and a written consent to use it.

References:

1. <https://euroqol.org/>

EQ-5D-5L:

Under each heading, please tick the ONE box that best describes your health TODAY.

1. MOBILITY

- I have no problems in walking about 
- I have slight problems in walking about 
- I have moderate problems in walking about 
- I have severe problems in walking about 
- I am unable to walk about 

1. SELF-CARE

- I have no problems washing or dressing myself 
- I have slight problems washing or dressing myself 
- I have moderate problems washing or dressing myself 
- I have severe problems washing or dressing myself 
- I am unable to wash or dress myself 

1. USUAL ACTIVITIES (e.g. work, study, housework, family or leisure activities)

- I have no problems doing my usual activities 
- I have slight problems doing my usual activities 
- I have moderate problems doing my usual activities 
- I have severe problems doing my usual activities 
- I am unable to do my usual activities 

1. PAIN / DISCOMFORT

- I have no pain or discomfort 
- I have slight pain or discomfort 
- I have moderate pain or discomfort 
- I have severe pain or discomfort 
- I have extreme pain or discomfort 

1. ANXIETY / DEPRESSION

- I am not anxious or depressed 
- I am slightly anxious or depressed 
- I am moderately anxious or depressed 
- I am severely anxious or depressed 
- I am extremely anxious or depressed 

2009 EuroQol Research Foundation. EQ-5D™ is a trade mark of the EuroQol Research Foundation. UK (English) v1.2

We would like to know how good or bad your health is TODAY.

This scale is numbered from 0 to 100.

100 means the best health you can imagine.

0 means the worst health you can imagine.

Please mark an X on the scale to indicate how your health is TODAY.

Now, write the number you marked on the scale in the box below.

YOUR HEALTH TODAY = _____


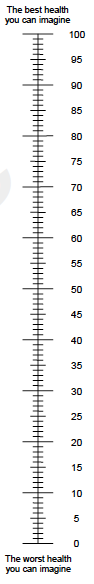


1. oQol Research Foundation. EQ-5D™ is a trade mark of the EuroQol Research Foundation. UK (English) v1.2
2. **Questionnaire on COVID-19 related symptoms at six months after infection**

| **Indicate if you present any of the following symptoms:** | **Yes** | **No** |
| --- | --- | --- |
| Fatigue |  |  |
| Dyspnoea |  |  |
| Dry cough |  |  |
| Sore throat |  |  |
| Productive cough |  |  |
| Rhinitis |  |  |
| Smell disorder |  |  |
| Decreased visual acuity |  |  |
| Conjunctival hyperaemia |  |  |
| Taste disorder |  |  |
| Inappetence |  |  |
| Diarrhoea |  |  |
| Myalgia |  |  |
| Arthralgia or Joint pain |  |  |
| Chest pain |  |  |
| Sicca syndrome |  |  |
| Raynaud syndrome |  |  |
| Skin lesion |  |  |
| Syncope |  |  |
| Dizziness |  |  |
| Headache |  |  |
